# Somatotopic functional connectivity gradients in the monkey and human striatum

**DOI:** 10.1162/IMAG.a.1421

**Published:** 2026-10-26

**Authors:** Naokazu Goda, Satomi Chiken, Daisuke Koketsu, Atsushi Nambu, Masaki Fukunaga

**Affiliations:** Section of Brain Function Information, National Institute for Physiological Sciences, Okazaki, Japan; Department of Physiological Sciences, SOKENDAI (The Graduate University for Advanced Studies), Okazaki, Japan; Core for Spin Life Sciences, National Institutes of Natural Sciences, Okazaki, Japan; Section of Multilayer Physiology, National Institute for Physiological Sciences, Okazaki, Japan; Division of Behavioral Development, National Institute for Physiological Sciences, Okazaki, Japan; Center for Research Collaboration, National Institute for Physiological Sciences, Okazaki, Japan

**Keywords:** somatotopy, functional connectivity, motor network, putamen, cross-species comparison

## Abstract

Understanding the topographic and functional organization of the striatum—a key brain structure involved in motor control, learning, and cognition—is crucial for elucidating the pathophysiology of the neurological and psychiatric disorders associated to it, and for developing targeted therapies. While recent magnetic resonance imaging (MRI) studies in humans and non-human primates have noninvasively characterized the brain-wide connectivity patterns of the striatum, its detailed somatotopic organization and the homology across species remains to be fully elucidated. We aimed to comprehensively characterize the somatotopic organization of the striatum in macaque monkeys based on functional connectivity patterns, and to assess the extent of homology or divergence of this organization between macaque monkeys and humans. Resting-state functional connectivity data were obtained from 11 anesthetized macaques using an ultra-high-field 7-tesla MRI scanner. Using two complementary approaches—seed-based analysis and connectopic mapping—we successfully delineated a distinct, regular volumetric somatotopic map within the macaque posterior putamen, a motor-related subregion of the striatum. This map exhibited a gradient encompassing the lower limb, trunk, upper limb, and face representations, predominantly oriented along the dorsal–ventral axis. The somatotopic gradient identified in macaques demonstrated a remarkable similarity with the corresponding gradient in the human posterior putamen, characterized using a publicly available resting-state functional MRI dataset. Furthermore, cross-species decoding analysis revealed that the somatotopy of the human posterior putamen could be accurately predicted using the anatomically aligned macaque somatotopic map obtained in this study as reference. In addition to this shared gradient organization, our seed-based analyses suggested non-gradient-like features in the human posterior putamen. These findings offer compelling evidence for the high evolutionary conservation of the major topographic organization in the primate posterior putamen, while pointing to species-specific divergence.

## 1 Introduction

The striatum, a key input structure of the basal ganglia, forms complex networks with both cortical and subcortical brain regions and plays crucial roles in motor control, learning, and cognition (Graybiel, 2008; Graybiel & Grafton, 2015). Recent advances in magnetic resonance imaging (MRI) techniques for analyzing structural and functional connectivity have enabled the noninvasive mapping of these networks in humans (Choi et al., 2012; Dosenbach et al., 2025; Draganski et al., 2008; Gordon et al., 2022; Greene et al., 2020; Jarbo & Verstynen, 2015; Kosakowski et al., 2024; Oldehinkel et al., 2022; Tian et al., 2020), as well as in non-human primates (NHPs) (Liu et al., 2021; Xia et al., 2023). These developments have provided new insights into the functional organization of the striatum and facilitated more direct comparisons of striatal architecture across species (Milardi et al., 2019).

Anatomical and electrophysiological studies in NHPs have established the somatotopic organization of the striatum and its topographic connectivity with the motor cortex (Alexander & DeLong, 1985; Flaherty & Graybiel, 1993; Künzle, 1975; Nambu, 2011; Nambu et al., 2002; Romanelli et al., 2005; Takada et al., 1998a, 1998b; Takara et al., 2011). Elucidating the detailed somatotopic map of the striatum is essential for understanding the pathophysiology of neurological disorders such as dystonia, which may involve the disorganization of striatal somatotopy (Delmaire et al., 2005). This map can also be used to develop effective therapeutic interventions, including the precise delivery of drugs or viral vectors. However, noninvasive assessment of this fundamental organization has not been sufficiently explored in NHPs. This also remains a significant challenge in humans (Marquis et al., 2019), hindering direct interspecies comparisons. Characterizing the degree of homology of the striatal somatotopy between humans and NHPs is necessary to confirm the validity of findings derived from NHPs for clinical applications.

In the present study, we aimed to noninvasively assess the somatotopic organization of the striatum in NHPs using resting-state functional MRI (rs-fMRI) and quantitatively compare the findings with corresponding data from humans. Eleven macaque monkeys (both male and female) underwent rs-fMRI under anesthesia using an ultra-high-field 7-tesla human MRI scanner. Given the well-established topographic anatomical connections between the striatum and the primary motor cortex (M1), we conducted seed-based functional connectivity analyses using multiple somatotopically defined seed regions within the M1 to examine the corresponding somatotopic organization within the striatum. To enable cross-species comparisons, the somatotopic organization of the human striatum was assessed using rs-fMRI data from the Midnight Scan Club (MSC) dataset (Gordon et al., 2017). We focused specifically on the posterior putamen, a motor-related subregion of the striatum, based on our results and previous literature, and performed direct comparisons of somatotopic topography using a novel cross-species decoding approach.

Beyond the seed-based analysis, we employed connectopic mapping—a data-driven approach that identifies spatial gradients in brain-wide functional connectivity profiles. This technique has been successfully applied to evaluate major topographies from rs-fMRI data, including somatotopy in the human M1 and primary somatosensory cortex (Haak et al., 2018; Ngo et al., 2021), and other functional gradients within the striatum in humans and NHPs (Manea et al., 2022; Marquand et al., 2017; Oldehinkel et al., 2022). We applied connectopic mapping for the first time to examine the somatotopic organization of the posterior putamen in both humans and NHPs.

## 2 Methods

### 2.1 Subjects

Eleven Japanese macaque monkeys (*Macaca fuscata*; 6 males, 5 females; aged 4–8 years; body weight 4.7–8.8 kg) participated in this study. Health status including body weight and food intake of the animals was monitored throughout the study. None of the animals underwent any surgical procedures prior to the rs-fMRI sessions, and they participated in other studies following their involvement in this experiment. All experiments were conducted in accordance with the National Institutes of Health “Guide for the Care and Use of Laboratory Animals,” and their protocols were approved by the local Institutional Animal Care and Use Committee.

### 2.2 MRI acquisition

MRI experiments were performed using a 7-tesla MRI (Magnetom 7T, Siemens) equipped with a macaque 24-channel receive coil and a volume transmission coil (Takashima Seisakusyo Co. Ltd.) (Autio et al., 2020; Goda et al., 2022). Manual shimming and B1 transmission field map acquisitions were performed at the beginning of each scanning session.

Anesthesia was induced via intramuscular injection of ketamine hydrochloride (5 mg/kg) and xylazine hydrochloride (0.6–0.8 mg/kg). The anesthesia was maintained with continuous intravenous infusion of propofol using a target-controlled infusion pump (TE-371, Terumo) to achieve a blood concentration of 3–7 μg/mL. Subjects were positioned supine, and their heads were immobilized using ear pads and a tooth bar within the coil. Body temperature was maintained with a blanket. Physiological parameters—including heart rate, arterial oxygen saturation, respiration rate, body temperature, and body movement—were continuously observed using MRI-compatible vital signs monitors (7500FO, Nonin; TSD110-MRI, FOTS100, BIOPAC) to confirm stabile conditions during the scanning (Supplementary Fig. S1).

#### 2.2.1 Structural data acquisition

Three-dimensional (3D) T1-weighted (T1w) structural images with a magnetization-prepared dual rapid gradient-echo (MP2RAGE) sequence (0.5 mm isotropic voxels; repetition time [TR] / echo time [TE], 2500/2.23 ms; inversion time, 800/2700 ms; generalized autocalibrating partially parallel acquisition (GRAPPA) acceleration factor, 3), and 3D T2-weighted (T2w) structural images with a sampling perfection with application-optimized contrasts using different flip angle evolutions (SPACE) sequence (0.5 mm isotropic; TR/TE, 3500/291 ms) were acquired for each subject. A pair of images with reversed phase- and frequency-encoding directions (one image for right-to-left and another for left-to-right phase-encoding) was acquired for each of the 3D T1w and T2w scans to enable geometric distortion correction. Two-dimensional (2D) T2w scans with a turbo spin-echo sequence (voxel size, 0.5 × 0.5 mm; slice thickness, 1.0 mm; 60 axial or oblique slices; TR/TE, 7500/85 ms), one for anterior-to-posterior (AP) and another for posterior-to-anterior (PA) phase-encoding direction, were also collected for structural-functional image registration.

#### 2.2.2 Rs-fMRI data acquisition

Rs-fMRI scans were initiated at least 90 minutes after ketamine and xylazine administration. Blood-oxygenation-level-dependent (BOLD) signals were acquired using a gradient-echo T2*-weighted (T2*w) echo-planar imaging (EPI) sequence (1.4 mm isotropic; 42 axial or oblique slices; TR/TE, 750/19.8 ms; multiband acceleration factor, 2; GRAPPA acceleration factor, 2). The voxel size was adopted for giving priority to increasing BOLD sensitivity. One or two pairs of rs-fMRI runs with AP and PA phase-encoding directions were acquired for each subject (1600 volumes [20 minutes] per run; two runs for six subjects, four runs for five subjects). Additionally, a pair of spin-echo EPIs (TR/TE, 4000/22 ms) with AP and PA phase-encoding was acquired.

### 2.3 Data analysis for macaque monkeys

MRI data were analyzed using the HCP-NHP pipeline (Autio et al., 2020; Donahue et al., 2016) with partial customizations (Goda et al., 2022), along with the HCP pipeline v4.3.0 (Glasser et al., 2013), Connectome Workbench v1.5.1 with wb_command (Marcus et al., 2011), FreeSurfer v5.3.0-HCP (Fischl, 2012), FSL v6.0.6 (Jenkinson et al., 2012), ANTs v2.4.2 (Avants et al., 2011), MRtrix3 (Tournier et al., 2019), DeepBet for macaque (Wang et al., 2021), BrainSpace (Vos de Wael et al., 2020), and MATLAB (MathWorks).

#### 2.3.1 Structural data preprocessing

The 3D structural images were corrected for Gibbs ringing artifacts using the mrdegibbs tool in MRtrix3. All structural images underwent gradient nonlinearity distortion correction using the HCP’s gradunwarp and geometrical distortion correction in the frequency-encoding directions using FSL’s topup. Bias field correction and brain extraction were initially performed using ANTs’s N4BiasFieldCorrection and DeepBet, respectively, and were further refined through the HCP-NHP structural pipeline. The images were subsequently processed with the pipeline for FreeSurfer’s surface reconstruction, sulcal depth estimation, and subcortical gray matter segmentation and parcellation. The images were then aligned to the HCP-NHP Yerkes19 (v1.2) macaque template through (both volume- and surface-based) nonlinear alignment.

For detailed delineation of subcortical areas, the SARM atlas from the NMT v2.0 (Hartig et al., 2021; Seidlitz et al., 2018) was nonlinearly aligned to the Yerkes19 template using ANTs. Specifically, the putamen and the adjacent claustrum were defined using the aligned SARM atlas. The M1 was defined using the CHARM atlas (Jung et al., 2021), which was nonlinearly aligned to the structural image of a representative subject (monkey FL) using ANTs and then mapped onto the cortical surface with the wb_command.

#### 2.3.2 Rs-fMRI data preprocessing

The rs-fMRI data were preprocessed using a customized HCP-NHP fMRIVolume pipeline. Briefly, the data were corrected for gradient nonlinearity distortion, geometric distortion, head motion, and bias field. The data were then rigidly registered to the T1w image in native space, using the distortion-corrected T2w image as an intermediate registration target, and nonlinearly aligned to the Yerkes19 space in 1-mm isotropic voxel resolution. The volumetric time series data were mapped onto the cortical surface, and converted to a CIFTI grayordinate format composed of cortical surface vertices and subcortical gray matter voxels (Glasser et al., 2013) using the HCP-NHP fMRISurface pipeline. During this process, cortical gray matter voxels were defined as cortical ribbon voxels and white matter voxels were removed from the subcortical data.

Independent component analysis (ICA)-based denoising (Salimi-Khorshidi et al., 2014) was applied to the preprocessed data (volumetric time series and CIFTI grayordinate time series) using the HCP ICA+FIX pipeline, with training data for macaques (Autio et al., 2020) included in the FSL. The denoised CIFTI grayordinate time series was band-pass filtered (0.008–0.09 Hz) (Yacoub et al., 2020), and spatially smoothed (geodesic smoothing for the cortical vertices and parcel-constrained volumetric smoothing for subcortical gray matter voxels; final full-width at half maximum [FWHM], 2 mm). To avoid contamination of the signals from the striatum with those from the neighboring claustrum through the spatial smoothing, the claustrum voxels were manually excluded from the CIFTI subcortical data prior to the smoothing.

#### 2.3.3 Seed-to-whole-brain functional connectivity analysis

Twenty seeds were defined on the anterior bank of the central sulcus within the left M1. These seeds were located at nearly equal sulcal depths and roughly equally spaced. Because no somatotopic cortical atlas for macaque monkeys was available, the somatotopic representations of the M1 seeds were defined based on conventional electrophysiological somatotopic mapping, which was conducted as a separate study. Detailed procedures for the mapping, including the confirmation of the mapping sites with additional MRI scanning, are described in the Supplementary Information.

A full functional connectivity matrix was computed from smoothed CIFTI grayordinate time series for each subject by using pairwise correlations (Fisher’s z) between all grayordinate time series (10,242 cortical vertices for each hemisphere plus 14,967 subcortical voxels) concatenated across runs (two runs for six subjects, and four runs for five). The functional connectivity matrix was then averaged across all subjects. The whole-brain seed-based functional connectivity map, showing connectivity with a specific M1 seed, was extracted from the individual or group-averaged full functional connectivity matrix and visualized using Connectome Workbench.

#### 2.3.4 Seed-to-ROI functional connectivity analysis

To investigate the seed-based functional connectivity within the striatum in more detail, a seed-to-ROI connectivity analysis was performed focusing on the posterior putamen. The posterior putamen ROI was defined as the postcommissural putamen (the segment of the putamen located posterior to the anterior commissure) in both hemispheres. For this analysis, non-smoothed volumetric time series denoised with ICA-FIX and band-pass filtered (0.008–0.09 Hz) were used to examine the functional connectivity profile with a higher spatial specificity compared to that allowed by the whole-brain analysis. In addition, the potential spurious influence of adjacent areas on the ROI was mitigated by following the methodology described in previous studies (Choi et al., 2012; Gordon et al., 2022; Greene et al., 2020). Specifically, for each subject, time series of the voxels in the adjacent regions (comprising all voxels in the claustrum and cortical ribbon voxels within 4 mm of the ROI) were extracted from the non-smoothed volumetric time series data. The first ten principal components of the time series of the adjacent regions computed using FSL fslmeants were regressed out from the ROI voxel time series using FSL fsl_glm. Then, correlation coefficients (Fisher’s z) between this adjusted ROI voxel time series and the M1 seed time series, which was extracted from the smoothed CIFTI grayordinate time series, were calculated to obtain the seed-to-ROI functional connectivity matrix (20 seeds × ROI voxels).

The seed-to-ROI functional connectivity matrix was subjected to a statistical test (permutation one-sample t-test, n = 11) using FSL PALM to identify ROI voxels where the group-averaged connectivity was significantly positive (p < 0.05, false discovery rate [FDR] corrected for multiple comparisons across all ROI voxels and seeds). The ROI voxels that were not significant for any seed condition were excluded from further seed-based analyses. The functional connectivity across all seed conditions was then compared for each ROI voxel to summarize the seed-based connectivity maps using a “winner-takes-all” approach. Prior to comparison, the map for each seed condition was normalized by its maximum value within the ROI to correct for potential connectivity biases across seed conditions, which could arise from differences in the signal-to-noise ratio at the seeds and other factors. A voxelwise “winner seed” map, representing the seed index with the strongest connectivity for each voxel, was then generated based on the normalized maps. Additionally, a voxelwise map of unnormalized functional connectivity with the winner seed was evaluated as an index of connectivity strength.

### 2.4 Data analysis for humans

#### 2.4.1 Dataset

The processed, non-smoothed volumetric time series and the smoothed CIFTI grayordinate time series of the MSC dataset (Gordon et al., 2017) were used in this study. The MSC dataset includes ten rs-fMRI runs (each lasting 30 minutes) per individual from ten adults (five women and five men, age range: 24–34 years). The rs-fMRI data were acquired using a 3T MRI scanner (Siemens TRIO) with a gradient-echo EPI sequence (TR/TE, 2200/27 ms, 4 mm isotropic voxels). The individuals were awake during the scans, with visual fixation on a white crosshair against a black background. Data from one individual (MSC08) was omitted from this study due to prolonged eye closures and head motions during the rs-fMRI runs (Gordon et al., 2017).

This dataset was chosen due to its resemblance to our macaque dataset in terms of spatial resolution and experimental strategy (small-N design). The voxel size was approximately comparable to that used for macaque monkeys, considering a 15-fold brain volume scaling factor (Hayashi et al., 2021). This dataset employed an intensive sampling approach from a relatively small number of individuals (2,733–7,667 volumes per individual from nine individuals after data exclusion), which was similar to our dataset (3,200 or 6,400 volumes per subject from 11 subjects).

The preprocessing steps applied for the processed rs-fMRI data included slice timing correction, intensity normalization, head motion correction, fieldmap-based distortion correction, alignment to Talairach atlas space, and resampling to 2-mm isotropic resolution. The time series then underwent denoising, including motion censoring, demeaning/detrending, temporal band-pass filtering (0.005–0.01 Hz), and a multiple regression of nuisance signals (principal components of white matter and ventricular signals, global mean signals, and motion estimates). The denoised volumetric time series were mapped onto the cortical surfaces and converted to the CIFTI grayordinate format, which was then spatially smoothed (geodesic smoothing for cortical surfaces and volumetric smoothing for subcortical volume, FWHM 6 mm). The motion-contaminated frames in the CIFTI grayordinate time series or non-smoothed volumetric time series as flagged by the temporal masks included in the dataset were then excluded. More details regarding the data acquisition and the preprocessing steps for this dataset have been described by Gordon et al. (2017).

In this study, an additional inter-individual alignment was performed following what was described by Greene et al. (2020). The non-smoothed volumetric time series were nonlinearly transformed from Talairach space to MNI space using ANTs with nearest neighbor interpolation. The CIFTI subcortical data were replaced with the MNI-transformed time series. Further, prior to the computation of a whole-brain, full functional connectivity matrix, the CIFTI subcortical data were edited to exclude the claustrum, as was the case for the macaque dataset, and then subjected to volumetric smoothing (FWHM 4 mm). The claustrum was defined based on the mask provided by Huang et al. (2025). The full functional connectivity matrix was obtained by correlating all CIFTI grayordinate time series (32,942 cortical vertices for each hemisphere plus 31,679 subcortical voxels) for each individual. These matrices were averaged across individuals and used for subsequent connectopic mapping analysis (Section 2.7).

#### 2.4.2 Seed-to-ROI functional connectivity analysis

Twenty M1 seeds were defined using the HCP-MMP 1.0 parcellation (Glasser et al., 2016), which includes five somatomotor subareas representing the face, eye, upper limb, trunk, and lower limb. The M1 seeds were located midway within the left area 4, spanning across these five somatomotor subareas.

As was the case in the analysis of the macaque dataset, the seed-to-ROI functional connectivity analysis was performed focusing on the posterior putamen. The posterior putamen ROI was defined using the Network 2 region of the 7-network striatal parcellation described by Choi et al. (2012), which has strong connectivity with the somatomotor areas in the cerebral cortex. For each individual, the ROI voxel time series was extracted from the non-smoothed volumetric time series. The first ten principal components of the voxel time series in the claustrum and the neighboring cortical regions (all claustrum voxels and cortical ribbon voxels within 9 mm in Talairach space from the ROI; Choi et al., 2012) were then regressed out from these extracted time series. The correlation coefficients between this adjusted ROI time series and the M1 seed time series, which were extracted from the smoothed CIFTI grayordinate time series, were calculated to obtain the seed-to-ROI functional connectivity matrix. The seed-to-ROI functional connectivity matrix was then analyzed in the same manner as in the case of the macaque dataset.

### 2.5 Cross-species anatomical alignment

To enable direct comparisons of the somatotopic topography in the macaque and human posterior putamen in the same coordinate space, cross-species anatomical alignment (Xia et al., 2023) was performed for the putamen. Using ANTs, the entire bilateral macaque putamen was nonlinearly aligned to the entire bilateral human putamen, defined according to the standard CIFTI parcellation, from which the claustrum voxels were manually excluded. Applying the interspecies transformation, the ROI functional connectivity maps for each seed and their summary maps (winner seed maps) were warped to the human putamen in MNI space.

### 2.6 Pattern classification decoding analysis

Multi-voxel pattern classification analysis was performed to quantitatively evaluate seed-dependent differences in the functional connectivity patterns of the posterior putamen ROI. The seed conditions were divided into ten classes, with two consecutive conditions per class (e.g., seeds #1 and #2 as class 1; seeds #3 and #4 as class 2, etc.), or into five classes, with four consecutive conditions per class. The performances for the 10-class and 5-class classifications for the macaque seed-to-ROI functional connectivity data or for the human data were evaluated using a simple correlation classification procedure (Haxby et al., 2001) with a leave-one-subject-out cross validation. The ROI functional connectivity maps for each seed from one subject were used as test samples (20 samples), and those from the remaining subjects of the same species were used as training samples. The template map was obtained by averaging the training samples for each of the classes. The class of the test sample was assigned to the class of the template that showed the highest correlation. The classification accuracy (number of correct classifications / numbers of test samples) and the confusion matrix were obtained for each subject, and then averaged across subjects. A bootstrap test was performed to assess whether the mean accuracy across subjects was significantly above the chance level.

Furthermore, to assess interspecies similarity in the topography of the seed-to-ROI functional connectivity, we evaluated how accurately the seed class for one species could be correctly classified using the data from another species as a training dataset. The correlation classification analysis was performed using the human ROI functional connectivity map for each seed and each individual as the test sample and the macaque ROI functional connectivity maps, which were warped to MNI space, as the training samples, and vice versa.

### 2.7 Connectopic mapping analysis

Connectopic mapping was performed for some ROIs using the functional connectivity matrix between an ROI and all other brain regions, extracted from the group-averaged, full functional connectivity matrix. This technique estimates the principal local topographic gradients of global connectivity patterns within an ROI, capturing smooth transitions by applying a dimensionality reduction method (spectral embedding) to high-dimensional connectivity data. Gradient components were computed using BrainSpace with default procedures. Briefly, the extracted functional connectivity matrix was converted to Pearson’s r, sparsified by thresholding each row at the 90 percentiles, and transformed into an affinity matrix using a normalized angle kernel. Diffusion mapping—a nonlinear spectral embedding algorithm—was then applied to derive the dominant connectivity gradients. The obtained gradient component was min–max normalized and visualized on the cortical surface as a pseudocolor map. This gradient component estimated within the ROI was then projected outside the ROI—onto cortical vertices and subcortical voxels—using a method described by Haak et al. (2018). Specifically, a gradient value of each external vertex or voxel was assigned to that of the ROI vertex or voxel with which exhibited the strongest functional connectivity.

A supplemental connectopic mapping analysis was performed for macaque data to assess the potential influence of preprocessing-induced spatial autocorrelation on the estimated gradient components (Watson & Andrews, 2023). Random Gaussian white noise time series, matched in mean and variance to the original fMRI data, were generated as control data using the make_random_timeseries.sh scripts provided by Watson and Andrews (2023). These synthetic datasets were processed identically to the real datasets, except for the exclusion of ICA-based denoising, to produce a control group-averaged functional connectivity matrix. Connectopic mapping was then applied to this control matrix using the same procedures as for the real data.

## 3 Results

### 3.1 Seed-based functional connectivity analysis of the striatum in macaque monkeys

We investigated the whole-brain functional connectivity using a set of somatotopically defined seed regions in the left M1, including representations of the lower limb, trunk, upper limb, and face (Fig. 1A). The somatotopic representations of these seed regions were confirmed through conventional electrophysiological mapping in one subject (Supplementary Fig. S2). When analyzing whole-brain functional connectivity using the M1 trunk region (seed #5), we observed robust bilateral functional connectivity across the frontal and parietal cortices, encompassing both motor and somatosensory areas (Fig. 1B, left; Supplementary Movie S1). At the subcortical level, the most prominent connectivity with the M1 trunk seed was observed in the bilateral putamen, a major component of the striatum (Fig. 1C, left).

**Fig. 1.**
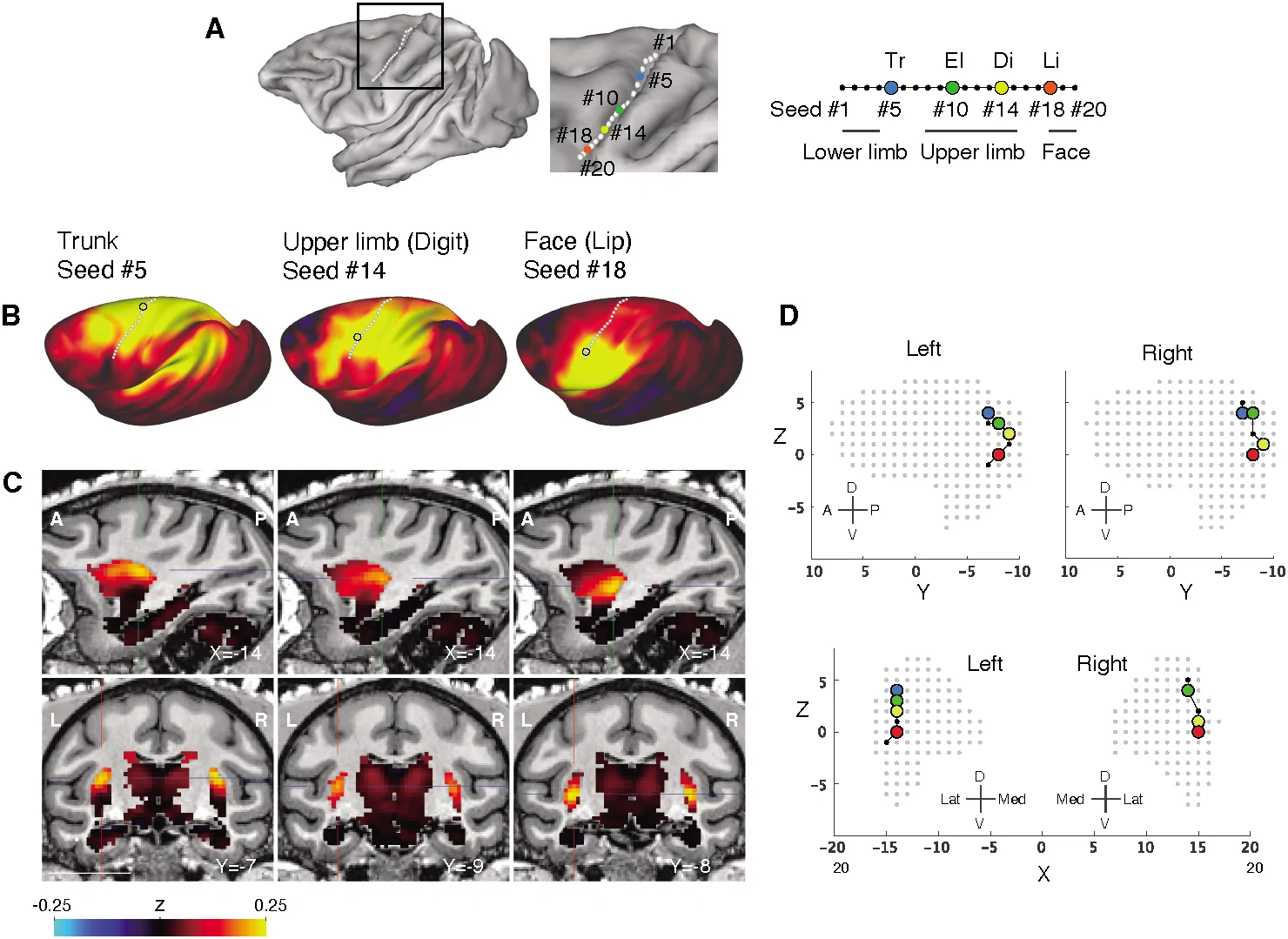
Seed-based functional connectivity maps in macaque monkeys. (A) Twenty seed regions in the M1 (left, middle) and the corresponding somatotopic representations (right). (B, C) Group-average functional connectivity maps with three somatotopically defined seeds in the left M1 (left: trunk seed; middle: upper limb seed; right: face seed). These maps are shown on the inflated left cortical surface (B) and within subcortical structures (C). The color map depicts unthresholded connectivity values (Fisher’s z). Black circles in (B) indicate seed locations. The sagittal and coronal planes in (C) were centered on the peak of the connectivity in the left striatum. The cortical surface and T1w structural volume are from one representative subject (monkey FL). (D) Spatial coordinates (Yerkes19 template space, with the origin at the anterior commissure) of the group-average functional connectivity peaks for all 20 seeds in the left and right striatum. Plots show the distribution of peaks on the sagittal (along the Y–Z axis, top) and coronal (along the X–Z axis, bottom) views. Gray dots indicate putamen voxels projected onto each view. Colored circles and black dots connected by lines indicate the locations of the peaks. Colors represented four seed conditions as shown in A, right. Scale bar, 20 mm in (C). A: anterior, D: dorsal, Di: digit, El: elbow, L: left, Lat: lateral, Li: lip, Med: medial, P: posterior, R: right, Tr: trunk, V: ventral.

As the M1 seed was moved from the posterodorsomedial trunk region to the anteroventrolateral upper limb region (digit, seed #14), the corresponding cortical functional connectivity map shifted ventrally (Fig. 1B, middle; Supplementary Movie S1). When the seed was placed further into the face region (lip, seed #18), the cortical functional connectivity map shifted even more ventrally (Fig. 1B, right; Supplementary Movie S1). Conversely, when the seed was moved dorsomedially from the trunk region (trunk, seed #5) to a putative lower limb representation (seed #1), the cortical functional connectivity map shifted dorsally (Supplementary Movie S1). In the subcortical region, the peak of functional connectivity shifted systematically in a dorsal-to-ventral direction within the putamen as the M1 seed progressed from the trunk to the upper limb and then to the face region (Fig. 1C). The dorsal-to-ventral gradient was observable in some individual results (Supplementary Fig. S3A). Analysis of the functional connectivity peak coordinates in the striatum (Fig. 1D) revealed that the peaks for the seeds spanning the lower limb, trunk, upper limb, and face regions also varied along the anterior–posterior axis. Specifically, the connectivity peak shifted from the anterodorsal region (lower limb) to the most posterior part (near the upper limb/face boundary), and then returned to the anteroventral region (face), tracing a curved trajectory along the boundary of the putamen (Fig. 1D). All connectivity peaks were located within the posterior putamen, posterior to the anterior commissure (Y = 0). Overall, these results provide clear evidence for somatotopically organized functional connectivity between the M1 and the bilateral posterior putamen. The observed dorsal-to-ventral somatotopic gradient in the bilateral posterior putamen aligns well with that described in previous anatomical and electrophysiological studies in macaque monkeys (Alexander & DeLong, 1985; Flaherty & Graybiel, 1993; Künzle, 1975; Nambu, 2011; Nambu et al., 2002; Romanelli et al., 2005; Takada et al., 1998a, 1998b; Takara et al., 2011).

### 3.2 Volumetric somatotopic map of the posterior putamen in macaque monkeys

Building on the above whole-brain results and previous anatomical evidence, we focused our analysis on the posterior putamen ROI to obtain a volumetric somatotopic map by summarizing seed-to-ROI functional connectivity patterns. For each ROI voxel that exhibited significantly positive connectivity with any of the 20 M1 seeds (p < 0.05, FDR-corrected), we identified the “winner” seed—defined as the seed with the strongest normalized connectivity to that voxel. Figure 2A displays the winner seed index map and the corresponding connectivity strength. The winner seed indices of the voxels with weaker connectivity strength are shown in lower-saturation colors to reflect their reduced contribution. The resulting maps revealed a clear dorsal-to-ventral gradient of lower limb–trunk–upper limb–face somatotopy, represented as a violet–blue–green-yellow-red gradient. This gradient extended across a large portion of the posterior putamen. Additionally, the maps confirmed a curved gradient along the posterior boundary of the putamen, with strong connectivity.

**Fig. 2.**
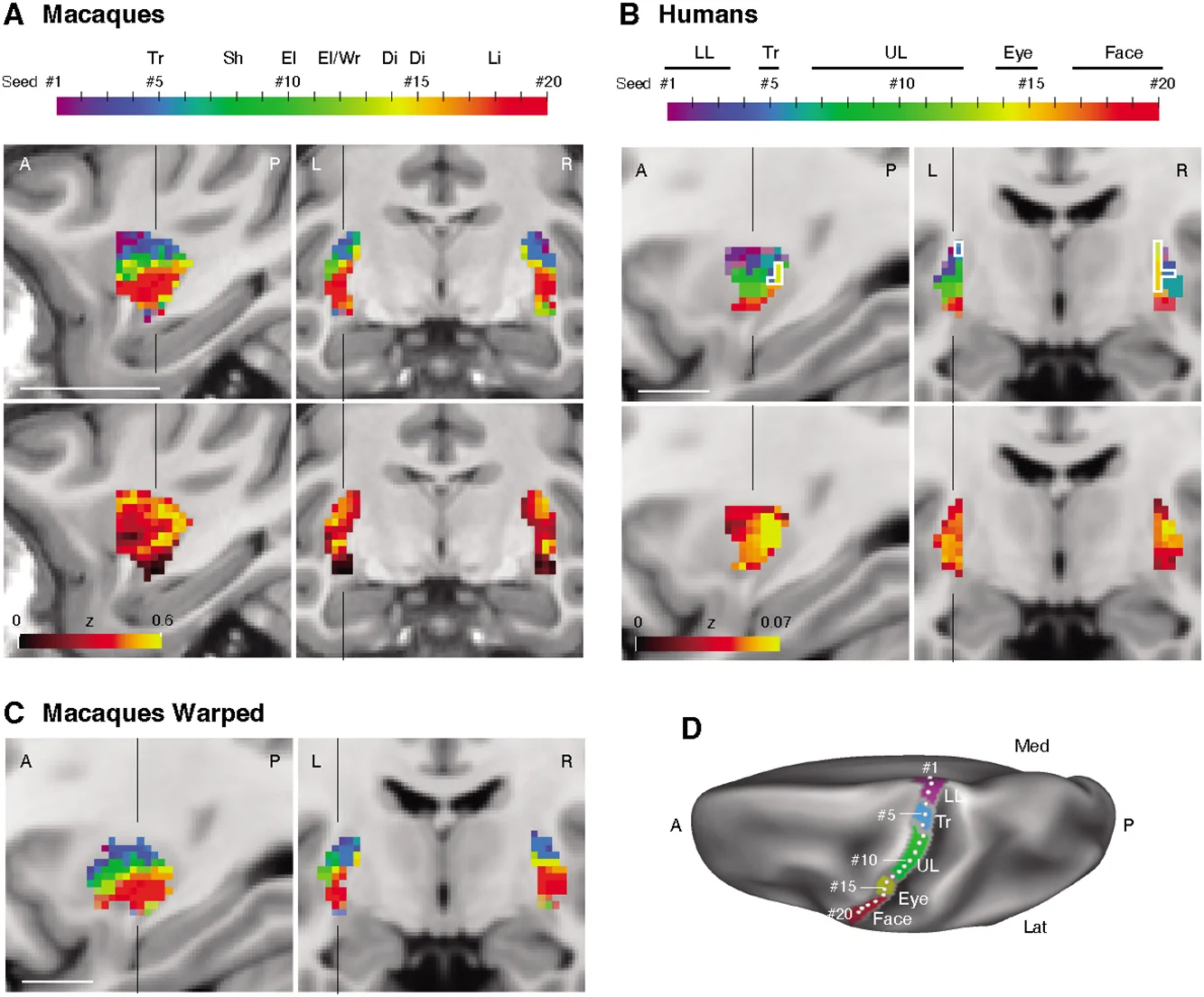
Volumetric somatotopic maps of the posterior putamen in macaque monkeys and humans derived from seed-to-ROI functional connectivity analysis. (A, B) Summary maps of seed-to-ROI functional connectivity analyses in macaque monkeys (A) and humans (B). In (A) and (B), the violet-blue-green-yellow-red color map in the top panels represents the seed index, ordered from lower limb to trunk, upper limb, and face. The middle panels show a map of the seed index with maximum normalized connectivity, represented by the color map in the top panel. The bottom panels show the strength of the connectivity (unnormalized Fisher’s z to the seed with maximum normalized connectivity). Voxel color in the middle panels was adjusted according to the connectivity strength shown in the bottom panels (100% saturation for the 100–75 percentile of Fisher’s z, 75% for the 75–50 percentile, 50% for the 50–25 percentile, and 25% for the 25–0 percentile). In the middle panel of (B), the voxels showing maximum normalized connectivity with the putative inter-effector seeds (seeds #5, #14, and #15) are enclosed by a white outline. (C) The map of the seed index with maximum normalized connectivity in the human space derived from cross-species anatomical transformation of the map in macaques (A, middle). (D) Seed locations in humans, mapped with the HCP-MMP 1.0 subarea parcellation. In (A), (B) and (C), only voxels showing significantly positive connectivity with at least one seed (p < 0.05, FDR corrected) are shown. Structural images are group-average T1w volumes from the Yerkes19 template for macaques (A) and the MNI template for humans (B, C). Scale bars, 20 mm in (A–C). Di: digit, El: elbow, Li: lip, LL: lower limb, Sh: shoulder, Tr: trunk, UL: upper limb.

The somatotopic gradient was consistently observed across subjects, even when they were analyzed in two separate subgroups (Supplementary Fig. S4). The connectivity maps from these two subgroups showed a high correlation (r = 0.745, p < 10^-6^, permutation test). Furthermore, we ruled out the possibility that the observed somatotopic gradient in the posterior putamen was merely reflecting the “inverted distance effect” (Salvador et al., 2005), a general tendency for connectivity to be stronger between physically close regions. Specifically, although all voxels in the macaque posterior putamen ROI were physically closest to seeds #16–#20 in Yerkes space, many of these voxels demonstrated stronger functional connectivity with the more distant seeds (#1–#15).

### 3.3 Cross-species comparison with humans

To compare the somatotopic organization of the putamen in macaque monkeys with that of humans, we performed a similar seed-based functional connectivity analysis using the MSC dataset (n = 9). We assessed the topography of the seed-based functional connectivity in the human posterior putamen by mapping the “winner” seed—defined as the seed with the strongest normalized connectivity—for each voxel. As in the macaque data, color saturation of the winner seed index was scaled according to connectivity strength to the winner seed, with lower saturation indicating weaker connectivity. The resulting map revealed a violet–blue–green-yellow-red gradient of strong connectivity extending from the dorsal to ventral regions of the posterior putamen (Fig. 2B), as was the case in macaques. This gradient reflected somatotopy spanning from lower limb to face, and aligned with the somatotopic patterns previously reported in the human posterior putamen using both resting-state and task-based fMRI (Choi et al., 2012; Kosakowski et al., 2024; Zeharia et al., 2015).

However, a closer look at the map revealed a disruption in the gradient mainly at the most posterior part of the posterior putamen ROI (enclosed by the white outline in Fig. 2B). In this and neighboring regions, voxels exhibiting the strongest connectivity with the spatially distant M1 trunk (blue) and eye (yellow) seeds lay adjacent to one another. This disruption likely reflects the strong functional connectivity between distant M1 inter-effector regions—specifically, the lower-limb/upper-limb inter-effector region and the upper-limb/face inter-effector region—as previously reported by Gordon et al. (2023). Consequently, voxels within the outlined area would exhibit comparable timeseries correlations with both the M1 trunk and eye seeds, which were mutually correlated, yielding the observed jumbled arrangement.

For more direct interspecies comparisons of the somatotopic topography, we performed nonlinear, anatomical alignment of the entire putamen across species. After the alignment, the warped macaque posterior putamen ROI covered 79 % of the human posterior putamen ROI. Even though the somatotopic representations of the 20 seeds were only approximately matched across species, the warped winner seed index map for macaques (Fig. 2C) was highly correlated with the map for humans (Fig. 2B) when compared over the intersection of the maps (r = 0.665, p < 10^-6^, permutation test). Notably, this strong cross-species correlation was driven even higher when adjusting for the human-specific disruption. Removing the voxels exhibiting the strongest connectivity with the putative inter-effector seeds (seeds #5 and #14–#15, within trunk and eye in human HCP-MMP 1.0 subarea parcellation, respectively) from the human map significantly improved correlation across species (r = 0.744), and this observed increase (Δ r = 0.079) was significantly larger than what would be expected by a random reduction of the number of voxels (p = 8.00 × 10^-6^, permutation test). These results indicate a substantial evolutionary conservation of the major connectivity topography between the M1 and the posterior putamen, alongside a cross-species divergence concerning the inter-effector representations.

### 3.4 Pattern classification decoding of functional connectivity patterns in the posterior putamen

Next, to further quantitatively evaluate the seed dependence of the functional connectivity patterns within the posterior putamen summarized above, we asked whether the seed condition, which reflected the somatotopic representation, was predictable from the functional connectivity patterns within the posterior putamen ROI. The classification accuracies for the 10 or 5 classes representing different somatotopy (Fig. 3A, top) were evaluated using a correlation classification procedure with a leave-one-subject-out cross validation. We found that the seed class was successfully predicted for both macaques and humans, with accuracies significantly exceeding chance level (Fig. 3A). These results demonstrate that the functional connectivity patterns within the ROI contain reliable information for distinguishing somatotopic representations. In the confusion matrices (Fig. 3B), which showed the assigned versus true class labels, the proportions of correctly classified samples (i.e., diagonal elements) in the macaque classification results were all high, indicating that the correct labels were assigned for all seed conditions almost uniformly. In contrast, for humans, the proportion of the diagonal elements were low for the classes including seeds #5 to #8, which corresponded to the somatotopic regions of the trunk and proximal upper limb. This suggests that the functional connectivity patterns for the seeds of those inter-effector somatotopic representations exhibit relatively variability across human individuals.

**Fig. 3.**
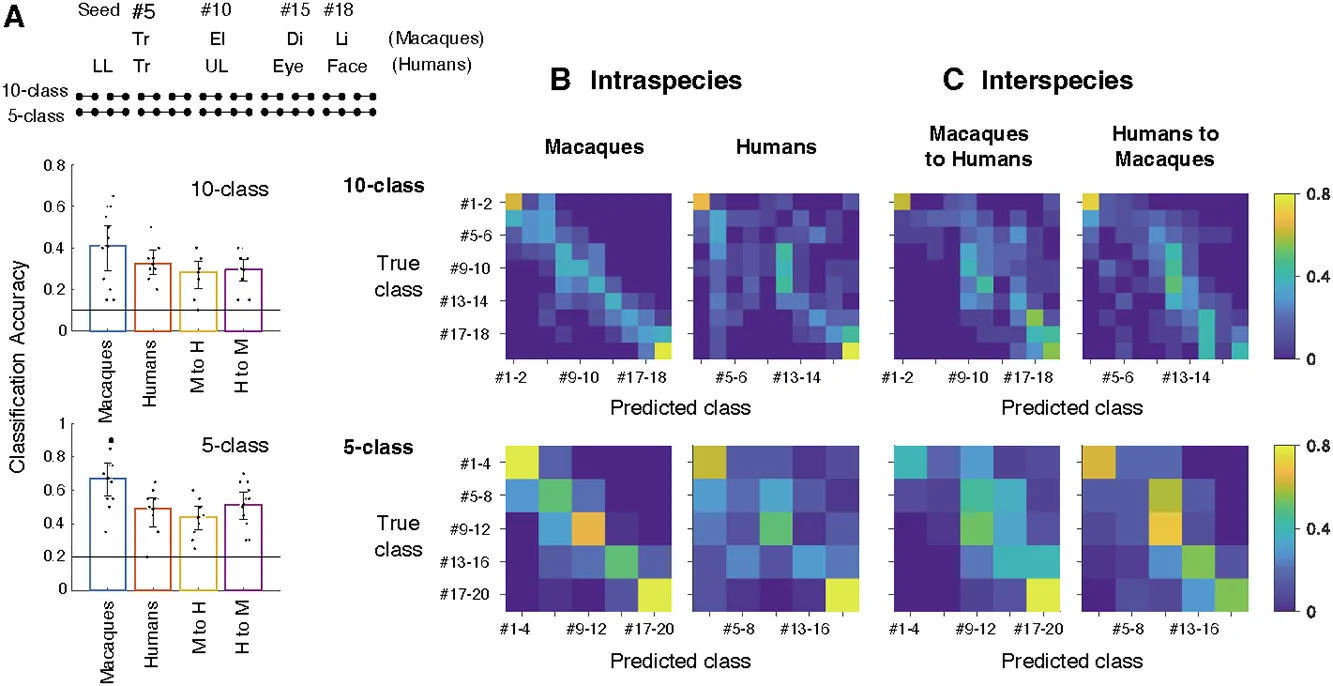
Pattern classification decoding analysis of the seed-based functional connectivity patterns in the posterior putamen. (A) 10 and 5 classes of the seed conditions together with the somatotopic representation in macaques and humans (top), mean accuracies for 10-class (middle) and 5-class (bottom) intraspecies classification obtained with the macaque and human data, and interspecies classification obtained with the human data as the test sample (Macaques to Humans, M to H), and with the macaque data as the test sample (Humans to Macaques, H to M). Error bars represent 95% confidence intervals evaluated by the bootstrap method. Each dot represents an individual result. (B, C) Confusion matrices for the 10-class (top) and 5-class (bottom) intraspecies classification (B), and interspecies classification (C). Rows and columns represent the true and predicted class labels, respectively. Row-normalized proportions are displayed with pseudo colors.

Given the high performance of the intraspecies classification, we then evaluated whether the seed class could be predicted using data from another species. The cross-species decoding results showed that the seed class of the functional connectivity pattern in the human posterior putamen ROI could be correctly predicted based on the correlation with those patterns in the macaque posterior putamen ROI, which was anatomically warped to the human MNI space (Fig. 3A, C, Macaques to Humans, M to H). The same was true when predicting the seed class of the macaque data using the human data (Fig. 3A, C, Humans to Macaques, H to M). The interspecies classification accuracies were comparable to those of the intraspecies classification. The confusion matrices were similar to the intraspecies human results, reflecting intra-individual variability for some seed conditions in humans, as mentioned above. These results provide further evidence of the high cross-species conservation of the major topographic organization, while supporting species-specific variations in the posterior putamen.

### 3.5 Connectopic mapping of the posterior putamen

To complement the seed-based analysis described above, we performed connectopic mapping to assess somatotopic organization within the posterior putamen. Previous studies in humans have demonstrated that the most dominant gradient of brain-wide functional connectivity patterns in the M1 reflects somatotopic organization (Haak et al., 2018). Even when multiple topographies overlap—such as eccentricity and polar angle representations in the primary visual cortex—this approach is expected to disentangle the overlapping topographies. To evaluate the applicability of this method to macaque rs-fMRI data, we first focused on the left M1. The first gradient component in the M1 is displayed on the cortical surface using a pseudocolor scale, where similar colors indicate similar brain-wide functional connectivity profiles (Fig. 4A). The color transitions gradually from violet to blue, green, yellow, and red along the central sulcus, in a posterodorsomedial to anteroventrolateral direction. This gradient reflects a smooth shift in connectivity patterns with other brain regions and corresponds well with the known lower limb–trunk–upper limb–face somatotopy. We confirmed that no such gradient was observed in a randomized time-series dataset, in which the mean and variance were matched to those of the real fMRI data (Supplementary Fig. S5), indicating that the somatotopic gradient was not an artifact generated during data preprocessing (Watson & Andrews, 2023).

**Fig. 4.**
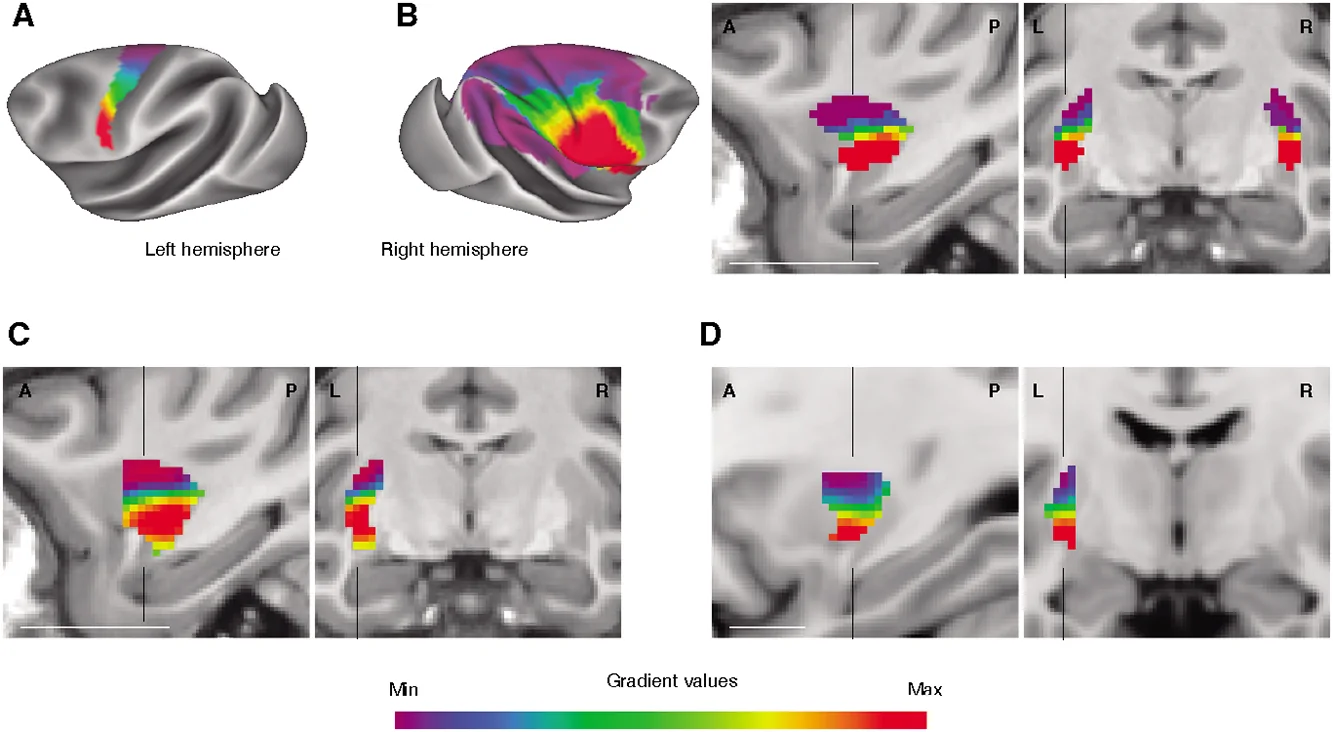
Volumetric somatotopic map in the posterior putamen derived from connectopic mapping analysis. (A) First functional connectivity gradient component within the left M1 of macaque monkeys, estimated using connectopic mapping. (B) Projection of the first gradient component within the left M1 to the right cortical hemisphere (left) and to the subcortical structures (middle and right). The projection map was thresholded according to maximum connectivity to the left M1 (Fisher’s z > 0.15). (C, D) First functional connectivity gradient within the left posterior putamen in macaques (C) and humans (D), estimated using connectopic mapping. The cortical surfaces in (A) and (B) are from monkey FL, and the structural image is a group-average T1w volume from the Yerkes19 template for macaques (B, C) and the MNI template for humans (D). Scale bars, 20 mm in (B–D).

To further investigate somatotopic organization in the posterior putamen—the primary focus of this analysis—we assigned a gradient value to each subcortical voxel as well as each vertex on the right cortical surface by projecting the gradient value (color-coded) from the left M1 vertex (Fig. 4B). The projection map was thresholded based on the strength of functional connectivity with the M1 vertices (Haak et al., 2018), using an arbitrary threshold of z = 0.15. The resulting map displayed a violet-blue-green-yellow-red gradient concordant with the lower limb–trunk–upper limb–face somatotopy, primarily along the dorsal–ventral axis of the posterior putamen. This projection-based somatotopic map demonstrated a strong correlation with the seed-based functional connectivity data shown in Figure 2A (r = 0.842, p < 10^-6^, permutation test).

Finally, we applied connectopic mapping directly to the left posterior putamen, using its functional connectivity with all other brain regions, for both macaque monkeys and humans. This analysis also revealed a somatotopic gradient (Fig. 4C, D). The resulting map for macaques (Fig. 4C) was highly consistent with both the seed-based map (Fig. 2A; r = 0.833, p < 10^-6^, permutation test) and the projection map based on the M1 connectopic mapping (Fig. 4B; r = 0.965, p < 10^-6^, permutation test). For humans, the gradient map (Fig. 4D) was highly correlated with the seed-based map of the left posterior putamen (Fig. 2B; r = 0.848, p < 10^-6^, permutation test), and even with the map in macaques warped to the human putamen (Fig. 2C; r = 0.899, p < 10^-6^, permutation test).

## 4 Discussion

In this study, we noninvasively examined the somatotopic organization of the striatum in macaque monkeys based on functional connectivity, with a particular focus on the M1, where distinct topographic connections are well established. Strong functional connectivity between the M1 and the bilateral posterior putamen was observed (Fig. 1, Supplementary Movie S1); therefore, our analysis focused on this region. Using both seed-based and connectopic mapping approaches, we demonstrated a clear somatotopic gradient in this region. The resulting somatotopic map closely aligned with findings from previous electrophysiological and anatomical tracer studies (Alexander & DeLong, 1985; Flaherty & Graybiel, 1993; Künzle, 1975; Nambu, 2011; Nambu et al., 2002; Takada et al., 1998a, 1998b; Takara et al., 2011). Unlike these gold-standard methods, our rs-fMRI–based approach is noninvasive and enables direct interspecies comparisons using the same methodology. Notably, when we applied the same seed-based analysis to human data, we identified analogous major somatotopic gradients in the human posterior putamen. Direct comparisons of the somatotopic gradients through cross-species anatomical alignment and decoding revealed that the topography of the gradient was remarkably homologous across species. The patterns of the gradients were highly correlated, and the seed conditions reflecting somatotopic representation could be accurately predicted from the functional connectivity patterns by correlating them with the group-averaged functional connectivity patterns from the other species. This striking interspecies homology of the major gradient suggests that the topographic organization, the fundamental functional architecture, in this region has been substantially conserved throughout evolution. Nevertheless, spatial disruptions were observed in the topography of connectivity with the M1 inter-effector regions, pointing to a potentially more complex non-gradient-like organization in humans. Another advantage is that this approach allows for comprehensive volumetric mapping of macro/meso-scale functional organization across the entire ROI. Our volumetric somatotopic map could serve as a valuable reference atlas for a wide range of studies, including both noninvasive and invasive investigations or therapies targeting specific somatotopic regions of the striatum. Potential near-future applications include stereotactic injection of viral vectors carrying dopamine-encoding genes or transplantation of dopamine-producing neurons derived from embryonic or induced pluripotent stem cells as therapeutic strategies for advanced Parkinson’s disease. The strong parallels of the somatotopic organization revealed in this study underscore the usefulness of macaque monkeys as preclinical models for clinical research or the development of targeted interventions.

Previous resting-state and task-related fMRI studies have identified multiple cortical networks encompassing motor and somatosensory cortices in both humans (Gordon et al., 2017) and NHPs (Hutchison et al., 2011; Sharma et al., 2019). Among these, one network typically includes the M1 face region, while another encompasses the M1 non-face regions—often referred to as the ventral and dorsal somatomotor networks, respectively. In humans, the dorsal somatomotor network is further subdivided into hand- and foot-related networks (Gordon et al., 2017). In our seed-based analysis, the cortical functional connectivity pattern diverged when the seed was moved across the boundary between the face and upper limb regions (Supplementary Movie S1), likely reflecting transitions between these somatomotor networks. In addition to these discrete transitions, we also observed a gradual shift in the cortical connectivity pattern as the seed was moved within each of the lower limb, upper limb, and face regions, suggesting continuous, fine-grained somatotopic organization (Supplementary Movie S1). Notably, these continuous changes in functional connectivity were not limited to the cerebral cortex but were also robust in the bilateral posterior putamen (Fig. 1C, D). This supports the fine-grained topographic connections from the M1 to the bilateral posterior putamen in macaques, including direct bilateral projections (Künzle, 1975; Takada et al., 1998b) and indirect pathway via the contralateral M1.

A recent study identified an additional network involving the M1 and the striatum in humans (Gordon et al., 2023). This network, called the somato-cognitive action network (SCAN), includes interconnected M1 inter-effector regions (such as the foot–hand and hand–mouth boundaries), the dorsal anterior cingulate cortex, and the dorsolateral putamen. While a homologous network between the M1 and the dorsal anterior cingulate cortex has been reported in monkeys (Gordon et al., 2023), the full extent and organization of the SCAN in NHPs remains unclear. In our group-averaged macaque data, the characteristic inter-effector connectivity pattern associated with the SCAN was not clearly evident (Supplementary Movie S1) consistent with the previous study (Gordon et al., 2023), although suggestive patterns were observed in some individual cases (Supplementary Fig. S3B). Additionally, the functional connectivity patterns of the posterior putamen with the M1 seeds at the boundary of the upper limb regions (Fig. 1D, Supplementary Movie S1, around seeds #6–#7 and #16–#17) exhibited only gradual transitions from adjacent effector-specific regions. On the other hand, in the human posterior putamen, an irregularity of the gradient (a jumbled arrangement) was observed, presumably due to the contribution of the SCAN. Intraspecies decoding results supported this observation. In humans, the classification accuracy for seeds #5–10, which encompassed the inter-effector representation, was low, likely owing to high irregularity and inter-individual variability. In contrast, no such seed-dependent variability was observed in macaques (Fig. 3B). These findings suggest that the contribution of the SCAN to the M1–posterior putamen functional connectivity is present in humans, whereas in macaques it is minimal, if present at all.

The functional connectivity between the M1 and subcortical regions other than the putamen, including the thalamus and cerebellum, was weak in macaques (Supplementary Movie S1). Therefore, robust somatotopic gradients of functional connectivity in these regions could not be confirmed. Somatotopic functional connectivity between the M1 and the globus pallidus, thalamus, and cerebellum has already been reported in humans (Buckner et al., 2011; Zeharia et al., 2015), suggesting the feasibility of similar investigations in NHPs. Evaluating these subcortical somatotopic representations from rs-fMRI data remains an important issue for future research. A key difference between human and macaque data is that human data were obtained in an awake state, whereas our macaque data were collected under anesthesia (Pagani et al., 2023). Future studies are required to investigate the effects of anesthetic conditions, as well as the optimal imaging parameters for subcortical structures, including spatial resolution, echo time, and imaging sequence (Marquis et al., 2019).

## Supplementary Material

Supplementary Material

Supplementary Movie

## Data Availability

Data to reproduce figures will be made available on BALSA database (https://balsa.wustl.edu/study/l09nK). The codes we used here are available via email upon request.

## References

[IMAG.a.1421-b1] Alexander, G. E., & DeLong, M. R. (1985). Microstimulation of the primate neostriatum. II. Somatotopic organization of striatal microexcitable zones and their relation to neuronal response properties. Journal of Neurophysiology, 53(6), 1417–1430. 10.1152/jn.1985.53.6.1417 4009227

[IMAG.a.1421-b2] Autio, J. A., Glasser, M. F., Ose, T., Donahue, C. J., Bastiani, M., Ohno, M., Kawabata, Y., Urushibata, Y., Murata, K., Nishigori, K., Yamaguchi, M., Hori, Y., Yoshida, A., Go, Y., Coalson, T. S., Jbabdi, S., Sotiropoulos, S. N., Kennedy, H., Smith, S., Van Essen, D., & Hayashi, T. (2020). Towards HCP-style macaque connectomes: 24-channel 3T multi-array coil, MRI sequences and preprocessing. NeuroImage, 215, 116800. 10.1016/j.neuroimage.2020.116800 PMC7116593 32276072

[IMAG.a.1421-b3] Avants, B. B., Tustison, N. J., Wu, J., Cook, P. A., & Gee, J. C. (2011). An open source multivariate framework for N-tissue segmentation with evaluation on public data. Neuroinformatics, 9(4), 381–400. 10.1007/s12021-011-9109-y PMC3297199 21373993

[IMAG.a.1421-b4] Buckner, R. L., Krienen, F. M., Castellanos, A., Diaz, J. C., & Yeo, B. T. T. (2011). The organization of the human cerebellum estimated by intrinsic functional connectivity. Journal of Neurophysiology, 106(5), 2322–2345. 10.1152/jn.00339.2011 PMC3214121 21795627

[IMAG.a.1421-b5] Choi, E. Y., Yeo, B. T. T., & Buckner, R. L. (2012). The organization of the human striatum estimated by intrinsic functional connectivity. Journal of Neurophysiology, 108, 2242–2263. 10.1152/jn.00270.2012 PMC3545026 22832566

[IMAG.a.1421-b6] Delmaire, C., Krainik, A., Tézenas du Montcel, Gerardin, S. E., Meunier, S., Mangin, J-F., Sangla, S., Garnero, L., Vidailhet, M., & Lehéricy, S. (2005). Disorganized somatotopy in the putamen of patients with focal hand dystonia. Neurology, 64(8), 1391–1396. 10.1212/01.WNL.0000158424.01299.76 15851729

[IMAG.a.1421-b7] Donahue, C. J., Sotiropoulos, S. N., Jbabdi, S., Hernandez-Fernandez, M., Behrens, T. E., Dyrby, T. B., Coalson, T., Kennedy, H., Knoblauch, K., Van Essen, D. C., & Glasser, M. F. (2016). Using diffusion tractography to predict cortical connection strength and distance: A quantitative comparison with tracers in the monkey. The Journal of Neuroscience, 36(25), 6758–6770. 10.1523/JNEUROSCI.0493-16.2016 PMC4916250 27335406

[IMAG.a.1421-b8] Dosenbach, N. U. F., Raichle, M. E., & Gordon, E. M. (2025). The brain’s action-mode network. Nature Reviews Neurosciences, 26, 158–168. 10.1038/s41583-024-00895-x 39743556

[IMAG.a.1421-b9] Draganski, B., Kherif, F., Klöppel, S., Cook, P. A., Alexander, D. C., Parker, G. J. M., Deichmann, R., Ashburner, J., & Frackowiak, R. S. J. (2008). Evidence for segregated and integrative connectivity patterns in the human basal ganglia. The Journal of Neuroscience, 28(28), 7143–7152. 10.1523/JNEUROSCI.1486-08.2008 PMC6670486 18614684

[IMAG.a.1421-b10] Fischl, B. (2012). FreeSurfer. NeuroImage, 62(2), 774–781. 10.1016/J.NEUROIMAGE.2012.01.021 PMC3685476 22248573

[IMAG.a.1421-b11] Flaherty, A. W., & Graybiel, A. M. (1993). Two input systems for body representations in the primate striatal matrix: Experimental evidence in the squirrel monkey. The Journal of Neuroscience, 13(3), 1120–1137. 10.1523/JNEUROSCI.13-03-01120.1993 PMC6576612 7680067

[IMAG.a.1421-b12] Glasser, M. F., Coalson, T. S., Robinson, E. C., Hacker, C. D., Harwell, J., Yacoub, E., Ugurbil, K., Andersson, J., Beckmann, C. F., Jenkinson, M., Smith, S. M., & Van Essen, D. C. (2016). A multi-modal parcellation of human cerebral cortex. Nature, 536(7615), 171–178. 10.1038/nature18933 PMC4990127 27437579

[IMAG.a.1421-b13] Glasser, M. F., Sotiropoulos, S. N., Wilson, J. A., Coalson, T. S., Fischl, B., Andersson, J. L., Xu, J., Jbabdi, S., Webster, M., Polimeni, J. R., Van Essen, D. C., & Jenkinson, M. (2013). The minimal preprocessing pipelines for the Human Connectome Project. NeuroImage, 80, 105–124. 10.1016/J.NEUROIMAGE.2013.04.127 PMC3720813 23668970

[IMAG.a.1421-b14] Goda, N., Hasegawa, T., Koketsu, D., Chiken, S., Kikuta, S., Sano, H., Kobayashi, K., Nambu, A., Sadato, N., & Fukunaga, M. (2022). Cerebro-cerebellar interactions in nonhuman primates examined by optogenetic functional magnetic resonance imaging. Cerebral Cortex Communications, 3(2), tgac022. 10.1093/texcom/tgac022 PMC9233902 35769971

[IMAG.a.1421-b15] Gordon, E. M., Chauvin, R. J., Van, A. N., Rajesh, A., Nielsen, A., Newbold, D. J., Lynch, C. J., Seider, N. A., Krimmel, S. R., Scheidter, K. M., Monk, J., Miller, R. L., Metoki, A., Montez, D. F., Zheng, A., Elbau, I., Madison, T., Nishino, T., Myers, M. J.,… Dosenbach, N. U. F. (2023). A somato-cognitive action network alternates with effector regions in motor cortex. Nature, 617, 351–359. 10.1038/s41586-023-05964-2 PMC10172144 37076628

[IMAG.a.1421-b16] Gordon, E. M., Laumann, T. O., Gilmore, A. W., Newbold, D. J., Greene, D. J., Berg, J. J., Ortega, M., Hoyt-Drazen, C., Gratton, C., Sun, H., Hampton, J. M., Coalson, R. S., Nguyen, A. L., McDermott, K. B., Shimony, J. S., Snyder, A. Z., Schlaggar, B. L., Petersen, S. E., Nelson, S. M., & Dosenbach, N. U. F. (2017). Precision functional mapping of individual human brains. Neuron, 95(4), 791–807.e7. 10.1016/j.neuron.2017.07.011 PMC5576360 28757305

[IMAG.a.1421-b17] Gordon, E. M., Laumann, T. O., Marek, S., Newbold, D. J., Hampton, J. M., Seider, N. A., Montez, D. F., Nielsen, A. M., Van, A. N., Zheng, A., Miller, R., Siegel, J. S., Kay, B. P., Snyder, A. Z., Greene, D. J., Schlaggar, B. L., Petersen, S. E., Nelson, S. M., & Dosenbach, N. U. F. (2022). Individualized functional subnetworks connect human striatum and frontal cortex. Cerebral Cortex, 32(13), 2868–2884. 10.1093/cercor/bhab387 PMC9247416 34718460

[IMAG.a.1421-b18] Graybiel, A. M. (2008). Habits, rituals, and the evaluative brain. Annual Review of Neuroscience, 31, 359–387. 10.1146/annurev.neuro.29.051605.112851 18558860

[IMAG.a.1421-b19] Graybiel, A. M., & Grafton, S. T. (2015). The striatum: Where skills and habits meet. Cold Spring Harbor Perspectives in Biology, *7*, a021691. https://doi:10.1101/cshperspect.a021691 10.1101/cshperspect.a021691 PMC4526748 26238359

[IMAG.a.1421-b20] Greene, D. J., Marek, S., Gordon, E. M., Siegel, J. S., Gratton, C., Laumann, T. O., Gilmore, A. W., Berg, J. J., Nguyen, A. L., Dierker, D., Van, A. N., Ortega, M., Newbold, D. J., Hampton, J. M., Nielsen, A. N., McDermott, K. B., Roland, J. L., Norris, S. A., Nelson, S. M., Snyder, A. Z., Bradley L., Schlaggar, B. L., Steven, E., Petersen, S. E., & Dosenbach, N. U. F. (2020). Integrative and network-specific connectivity of the basal ganglia and thalamus defined in Individuals. Neuron, 105(4), 742–758.e6. 10.1016/j.neuron.2019.11.012 PMC7035165 31836321

[IMAG.a.1421-b21] Haak, K. V., Marquand, A. F., & Beckmann, C. F. (2018). Connectopic mapping with resting-state fMRI. NeuroImage, 170, 83–94. 10.1016/J.NEUROIMAGE.2017.06.075 28666880

[IMAG.a.1421-b22] Hartig, R., Glen, D., Jung, B., Logothetis, N. K., Paxinos, G., Garza-Villarreal, E. A., Messinger, A., & Evrard, H. C. (2021). The subcortical atlas of the rhesus macaque (SARM) for neuroimaging. NeuroImage, 235, 117996. 10.1016/j.neuroimage.2021.117996 33794360

[IMAG.a.1421-b23] Haxby, J. V., Gobbini, M. I., Furey, M. L., Ishai, A., Schouten, J. L., & Pietrini, P. (2001). Distributed and overlapping representations of faces and objects in ventral temporal cortex. Science, 293, 2425–2430. 10.1126/science.1063736 11577229

[IMAG.a.1421-b24] Hayashi, T., Hou, Y., Glasser, M. F., Autio, J. A., Knoblauch, K., Inoue-Murayama, M., Coalson, T., Yacoub, E., Smith, S., Kennedy, H., & van Essen, D. C. (2021). The nonhuman primate neuroimaging and neuroanatomy project. NeuroImage, 229, 117726. 10.1016/J.NEUROIMAGE.2021.117726 PMC8079967 33484849

[IMAG.a.1421-b25] Huang, C. H. L., Stewart, B. W., Liu, C. Y., Phylactou, P., Mathur, B. N., & Seminowicz, D. A. (2025). The human claustrum activates across multiple cognitive control tasks. The European Journal of Neuroscience, 62(9), e70318. 10.1111/ejn.70318 PMC12617337 41235903

[IMAG.a.1421-b26] Hutchison, R. M., Leung, L. S., Mirsattari, S. M., Gati, J. S., Menon, R. S., & Everling, S. (2011). Resting-state networks in the macaque at 7 T. NeuroImage, 56(3), 1546–1555. 10.1016/J.NEUROIMAGE.2011.02.063 21356313

[IMAG.a.1421-b27] Jarbo, K., & Verstynen, T. D. (2015). Converging structural and functional connectivity of orbitofrontal, dorsolateral prefrontal, and posterior parietal cortex in the human striatum. The Journal of Neuroscience, 35(9), 3865–3878. 10.1523/JNEUROSCI.2636-14.2015 PMC4461697 25740516

[IMAG.a.1421-b28] Jenkinson, M., Beckmann, C. F., Behrens, T. E. J., Woolrich, M. W., & Smith, S. M. (2012). FSL. NeuroImage, 62(2), 782–790. 10.1016/J.NEUROIMAGE.2011.09.015 21979382

[IMAG.a.1421-b29] Jung, B., Taylor, P. A., Seidlitz, J., Sponheim, C., Perkins, P., Ungerleider, L. G., Glen, D., & Messinger, A. (2021). A comprehensive macaque fMRI pipeline and hierarchical atlas. NeuroImage, 235, 117997. 10.1016/j.neuroimage.2021.117997 PMC9272767 33789138

[IMAG.a.1421-b30] Kosakowski, H. L., Saadon-Grosman, N., Du, J., Eldaief, M. C., & Buckner, R. L. (2024). Human striatal association megaclusters. Journal of Neurophysiology, 131(6), 1083–1100. 10.1152/jn.00387.2023 PMC11383613 38505898

[IMAG.a.1421-b31] Künzle, H. (1975). Bilateral projections from precentral motor cortex to the putamen and other parts of the basal ganglia. An autoradiographic study in Macaca fascicularis. Brain Research, 88(2), 195–209. 10.1016/0006-8993(75)90384-4 50112

[IMAG.a.1421-b32] Liu, X., Eickhoff, S. B., Caspers, S., Wu, J., Genon, S., Hoffstaedter, F., Mars, R. B., Sommer, I. E., Eickhoff, C. R., Chen, J., Jardri, R., Reetz, K., Dogan, I., Aleman, A., Kogler, L., Gruber, O., Caspers, J., Mathys, C., & Patil, K. R. (2021). Functional parcellation of human and macaque striatum reveals human-specific connectivity in the dorsal caudate. NeuroImage, 235, 118006. 10.1016/J.NEUROIMAGE.2021.118006 PMC8214073 33819611

[IMAG.a.1421-b33] Manea, A. M., Zilverstand, A., Ugurbil, K., Heilbronner, S. R., & Zimmermann, J. (2022). Intrinsic timescales as an organizational principle of neural processing across the whole rhesus macaque brain. ELife, 11, e75540. 10.7554/eLife.75540 PMC8923667 35234612

[IMAG.a.1421-b34] Marcus, D. S., Harwell, J., Olsen, T., Hodge, M., Glasser, M. F., Prior, F., Jenkinson, M., Laumann, T., Curtiss, S. W., & Van Essen, D. C. (2011). Informatics and data mining tools and strategies for the human connectome project. Frontiers in Neuroinformatics, 5, 4. 10.3389/fninf.2011.00004 PMC3127103 21743807

[IMAG.a.1421-b35] Marquand, A. F., Haak, K. V., & Beckmann, C. F. (2017). Functional corticostriatal connection topographies predict goal-directed behaviour in humans. Nature Human Behaviour, 1(8), 0146. 10.1038/s41562-017-0146 PMC5549843 28804783

[IMAG.a.1421-b36] Marquis, R., Muller, S., Lorio, S., Rodriguez-Herreros, B., Melie-Garcia, L., Kherif, F., Lutti, A., & Draganski, B. (2019). Spatial resolution and imaging encoding fMRI settings for optimal cortical and subcortical motor somatotopy in the human brain. Frontiers in Neuroscience, 13, 571. 10.3389/fnins.2019.00571 PMC6579882 31244595

[IMAG.a.1421-b37] Milardi, D., Quartarone, A., Bramanti, A., Anastasi, G., Bertino, S., Basile, G. A., Buonasera, P., Pilone, G., Celeste, G., Rizzo, G., Bruschetta, D., & Cacciola, A. (2019). The cortico-basal ganglia-cerebellar network: Past, present and future perspectives. Frontiers in Systems Neuroscience, 13, 61. 10.3389/fnsys.2019.00061 PMC6831548 31736719

[IMAG.a.1421-b38] Nambu, A. (2011). Somatotopic organization of the primate basal ganglia. Frontiers in Neuroanatomy, 5, 26. 10.3389/fnana.2011.00026 PMC3082737 21541304

[IMAG.a.1421-b39] Nambu, A., Kaneda, K., Tokuno, H., & Takada, N. (2002). Organization of corticostriatal motor inputs in monkey putamen. Journal of Neurophysiology, 88, 1830–1842. 10.1152/jn.2002.88.4.1830 12364509

[IMAG.a.1421-b40] Ngo, G. N., Haak, K. V., Beckmann, C. F., & Menon, R. S. (2021). Mesoscale hierarchical organization of primary somatosensory cortex captured by resting-state-fMRI in humans. NeuroImage, 235, 118031. 10.1016/J.NEUROIMAGE.2021.118031 PMC7618944 33836270

[IMAG.a.1421-b41] Oldehinkel, M., Llera, A., Faber, M., Huertas, I., Buitelaar, J. K., Bloem, B. R., Marquand, A. F., Helmich, R. C., Haak, K. V., & Beckmann, C. F. (2022). Mapping dopaminergic projections in the human brain with resting-state fMRI. ELife, 11, e71846. 10.7554/ELIFE.71846 PMC8843090 35113016

[IMAG.a.1421-b42] Pagani, M., Gutierrez-Barragan, D., de Guzman, A. E., Xu, T., & Gozzi, A. (2023). Mapping and comparing fMRI connectivity networks across species. Communications Biology, 6, 1238. 10.1038/s42003-023-05629-w PMC10703935 38062107

[IMAG.a.1421-b43] Romanelli, P., Esposito, V., Schaal, D. W., & Heit, G. (2005). Somatotopy in the basal ganglia: Experimental and clinical evidence for segregated sensorimotor channels. Brain Research Reviews, 48(1), 112–128. 10.1016/J.BRAINRESREV.2004.09.008 15708631

[IMAG.a.1421-b44] Salimi-Khorshidi, G., Douaud, G., Beckmann, C. F., Glasser, M. F., Griffanti, L., & Smith, S. M. (2014). Automatic denoising of functional MRI data: Combining independent component analysis and hierarchical fusion of classifiers. NeuroImage, 90, 449–468. 10.1016/J.NEUROIMAGE.2013.11.046 PMC4019210 24389422

[IMAG.a.1421-b45] Salvador, R., Suckling, J., Coleman, M. R., Pickard, J. D., Menon, D., & Bullmore, E. (2005). Neurophysiological architecture of functional magnetic resonance images of human brain. Cerebral Cortex, 15(9), 1332–2342. 10.1093/cercor/bhi016 15635061

[IMAG.a.1421-b46] Seidlitz, J., Sponheim, C., Glen, D., Ye, F. Q., Saleem, K. S., Leopold, D. A., Ungerleider, L., & Messinger, A. (2018). A population MRI brain template and analysis tools for the macaque. NeuroImage, 170, 121–131. 10.1016/J.NEUROIMAGE.2017.04.063 PMC5660669 28461058

[IMAG.a.1421-b47] Sharma, S., Mantini, D., Vanduffel, W., & Nelissen, K. (2019). Functional specialization of macaque premotor F5 subfields with respect to hand and mouth movements: A comparison of task and resting-state fMRI. NeuroImage, 191, 441–456. 10.1016/J.NEUROIMAGE.2019.02.045 30802514

[IMAG.a.1421-b48] Takada, M., Tokuno, H., Nambu, A., & Inase, M. (1998a). Corticostriatal projections from the somatic motor areas of the frontal cortex in the macaque monkey: Segregation versus overlap of input zones from the primary motor cortex, the supplementary motor area, and the premotor cortex. Experimental Brain Research, 120, 114–128. 10.1007/s002210050384 9628410

[IMAG.a.1421-b49] Takada, M., Tokuno, H., Nambu, A., & Inase, M. (1998b). Corticostriatal input zones from the supplementary motor area overlap those from the contra- rather than ipsilateral primary motor cortex. Brain Research, 791, 335–340. 10.1016/S0006-8993(98)00198-X 9593979

[IMAG.a.1421-b50] Takara, S., Hatanaka, N., Takada, M., & Nambu, A. (2011). Differential activity patterns of putaminal neurons with inputs from the primary motor cortex and supplementary motor area in behaving monkeys. Journal of Neurophysiology, 106(3), 1203–1217. 10.1152/jn.00768.2010 21653711

[IMAG.a.1421-b51] Tian, Y., Margulies, D. S., Breakspear, M., & Zalesky, A. (2020). Topographic organization of the human subcortex unveiled with functional connectivity gradients. Nature Neuroscience, 23(11), 1421–1432. 10.1038/s41593-020-00711-6 32989295

[IMAG.a.1421-b52] Tournier, J. D., Smith, R., Raffelt, D., Tabbara, R., Dhollander, T., Pietsch, M., Christiaens, D., Jeurissen, B., Yeh, C. H., & Connelly, A. (2019). MRtrix3: A fast, flexible and open software framework for medical image processing and visualisation. NeuroImage, 202, 116137. 10.1016/J.NEUROIMAGE.2019.116137 31473352

[IMAG.a.1421-b53] Vos de Wael, R., Benkarim, O., Paquola, C., Lariviere, S., Royer, J., Tavakol, S., Xu, T., Hong, S. J., Langs, G., Valk, S., Misic, B., Milham, M., Margulies, D., Smallwood, J., & Bernhardt, B. C. (2020). BrainSpace: A toolbox for the analysis of macroscale gradients in neuroimaging and connectomics datasets. Communications Biology, 3, 103. 10.1038/s42003-020-0794-7 PMC7058611 32139786

[IMAG.a.1421-b54] Wang, X., Li, X. H., Cho, J. W., Russ, B. E., Rajamani, N., Omelchenko, A., Ai, L., Korchmaros, A., Sawiak, S., Benn, R. A., Garcia-Saldivar, P., Wang, Z., Kalin, N. H., Schroeder, C. E., Craddock, R. C., Fox, A. S., Evans, A. C., Messinger, A., Milham, M. P., & Xu, T. (2021). U-net model for brain extraction: Trained on humans for transfer to non-human primates. NeuroImage, 235, 118001. 10.1016/J.NEUROIMAGE.2021.118001 PMC8529630 33789137

[IMAG.a.1421-b55] Watson, D. M., & Andrews, T. J. (2023). Connectopic mapping techniques do not reflect functional gradients in the brain. NeuroImage, 277, 120228. 10.1016/J.NEUROIMAGE.2023.120228 37339700

[IMAG.a.1421-b56] Xia, X., Zeng, X., Gao, F., & Yuan, Z. (2023). Mapping cross-species connectome atlas of human and macaque striatum. Cerebral Cortex, 33(12), 7518–7530. 10.1093/cercor/bhad057 PMC10267647 36928317

[IMAG.a.1421-b57] Yacoub, E., Grier, M. D., Auerbach, E. J., Lagore, R. L., Harel, N., Adriany, G., Zilverstand, A., Hayden, B. Y., Heilbronner, S. R., Uğurbil, K., & Zimmermann, J. (2020). Ultra-high field (10.5 T) resting state fMRI in the macaque. NeuroImage, 223, 117349. 10.1016/J.NEUROIMAGE.2020.117349 PMC7745777 32898683

[IMAG.a.1421-b58] Zeharia, N., Hertz, U., Flash, T., & Amedi, A. (2015). New whole-body sensory-motor gradients revealed using phase-locked analysis and verified using multivoxel pattern analysis and functional connectivity. The Journal of Neuroscience, 35(7), 2845–2859. 10.1523/JNEUROSCI.4246-14.2015 PMC6605596 25698725

