# Supplementary material for: Somatotopic functional connectivity gradients in the monkey and human striatum
Source: Imaging Neurosci (Camb). 2026 Oct 26;4:IMAG.a.1421. doi: 10.1162/IMAG.a.1421 (PMC13710930; doi:10.1162/IMAG.a.1421)
Supplement: Supplementary file 1 [file IMAG.a.1421_supp.pdf]

Supplementary Information

Supplementary text for the monkey M1 mapping

After completing the structural and rs-fMRI scans, one monkey (FL) underwent conventional electrophysiological somatotopic mapping of the M1. This mapping was conducted as a separate optogenetic intracortical microstimulation (ICMS) study (Goda et al., 2022; Watanabe et al., 2020). Briefly, the somatotopic representation of various M1 sites was determined based on body part movements evoked by applying electrical ICMS. Nine distinct sites were identified, including representations for the lips, digits, wrist/elbow, elbow, shoulder/elbow, and trunk. Based on this mapping, an adeno-associated virus (AAV) vector carrying channelrhodopsin-2 was injected into the functionally defined somatotopic regions. Detailed procedures are described as follows.

Surgery

Anesthesia was induced with ketamine hydrochloride (5–8 mg/kg body weight, intramuscular [i.m.]) and xylazine hydrochloride (0.5–1 mg/kg, i.m.), followed by intravenous administration of propofol (6–8 μg/mL blood concentration). After the skin incision, the skull was widely exposed, and the periosteum was completely removed. Small polycarbonate screws were anchored to the skull, and the exposed skull and screws were completely covered with transparent acrylic resin. A polyvinyl chloride base for head fixation was then attached and secured with acrylic resin. All surgical procedures were performed under aseptic conditions, with continuous monitoring of arterial oxygen saturation and heart rate. Anesthesia depth was evaluated based on heart rate and body movements. Additional anesthetic agents were administered when required. Antibiotics and analgesics were administered intramuscularly postoperatively.

M1 mapping and viral vector injection

After full recovery from surgery, the monkey was securely seated quietly in a monkey chair, with its head painlessly fixed in a stereotaxic frame using plastic pipes attached to a polyvinyl chloride base. The skull over the left central sulcus was then removed under anesthesia with ketamine hydrochloride (8 mg/kg, i.m.). The regions of the M1 representing the face, upper limb, trunk, and lower limb were identified through electrophysiological mapping (Goda et al., 2022; Nambu et al., 2000; Watanabe et al., 2020). Briefly, a glass-coated tungsten microelectrode (380-120605-00, 0.5 MΩ, Alpha

Omega) was inserted perpendicular to the cortical surface after lidocaine application. Neuronal responses were assessed through somatosensory stimulation. Electrical ICMS was then performed using monopolar stimulation (12 cathodal pulses, 0.2-ms duration, 333 Hz, <50 μA strength), with movements in the right side of the body being observed. Based on the mapping, an AAV vector carrying channelrhodopsin-2 was injected into the identified somatotopic region of the M1 (Watanabe et al., 2020; Goda et al., 2022). After the injections, the exposed area was completely covered with acrylic resin.

High-resolution structural MRI after M1 mapping

The tracks of the AAV injections were identified through an additional in vivo MRI session. High-resolution 3D T2*w structural images were acquired using a gradient-recalled echo (GRE) sequence (0.3 mm isotropic voxels, 50 coronal slices, TR/TE, 1200/14.2 ms), along with whole-brain T2w images. Scanning was performed using the same scanner and procedures described in Section 2.2, except that a cylindrical 8-channel receive coil (Takashima Seisakusyo Co., Ltd.) was used, and the monkey's head was stabilized using a headpost. The T2*w and T2w images were distortion-corrected as described in Section 2.3.1, and nonlinearly aligned to the structural images of the same monkey (FL) used for the rs-fMRI data analysis. Alignment was performed using ANTs, with the whole-brain T2w image serving as an intermediate registration target. The somatotopic representations of the M1 seeds were then identified using the injection tracks visible in the T2*w images as landmarks (Fig. S2).

Supplementary References

Goda, N., Hasegawa, T., Koketsu, D., Chiken, S., Kikuta, S., Sano, H., Kobayashi, K., Nambu, A., Sadato, N., Fukunaga, M. (2022). Cerebro-cerebellar interactions in nonhuman primates examined by optogenetic functional magnetic resonance imaging. *Cereb Cortex Commun 3*, tgac022.

Nambu, A., Tokuno, H., Hamada, I., Kita, H., Imanishi, M., Akazawa, T., Ikeuchi, Y., Hasegawa, N. (2000). Excitatory cortical inputs to pallidal neurons via the subthalamic nucleus in the monkey. *J Neurophysiol 84*, 289–300.

Watanabe, H., Sano, H., Chiken, S., Kobayashi, K., Fukata, Y., Fukata, M., Mushiake, H., Nambu, A. (2020). Forelimb movements evoked by optogenetic stimulation of the macaque motor cortex. *Nat Commun 11*, 3253.

Supplementary Movies and Figure

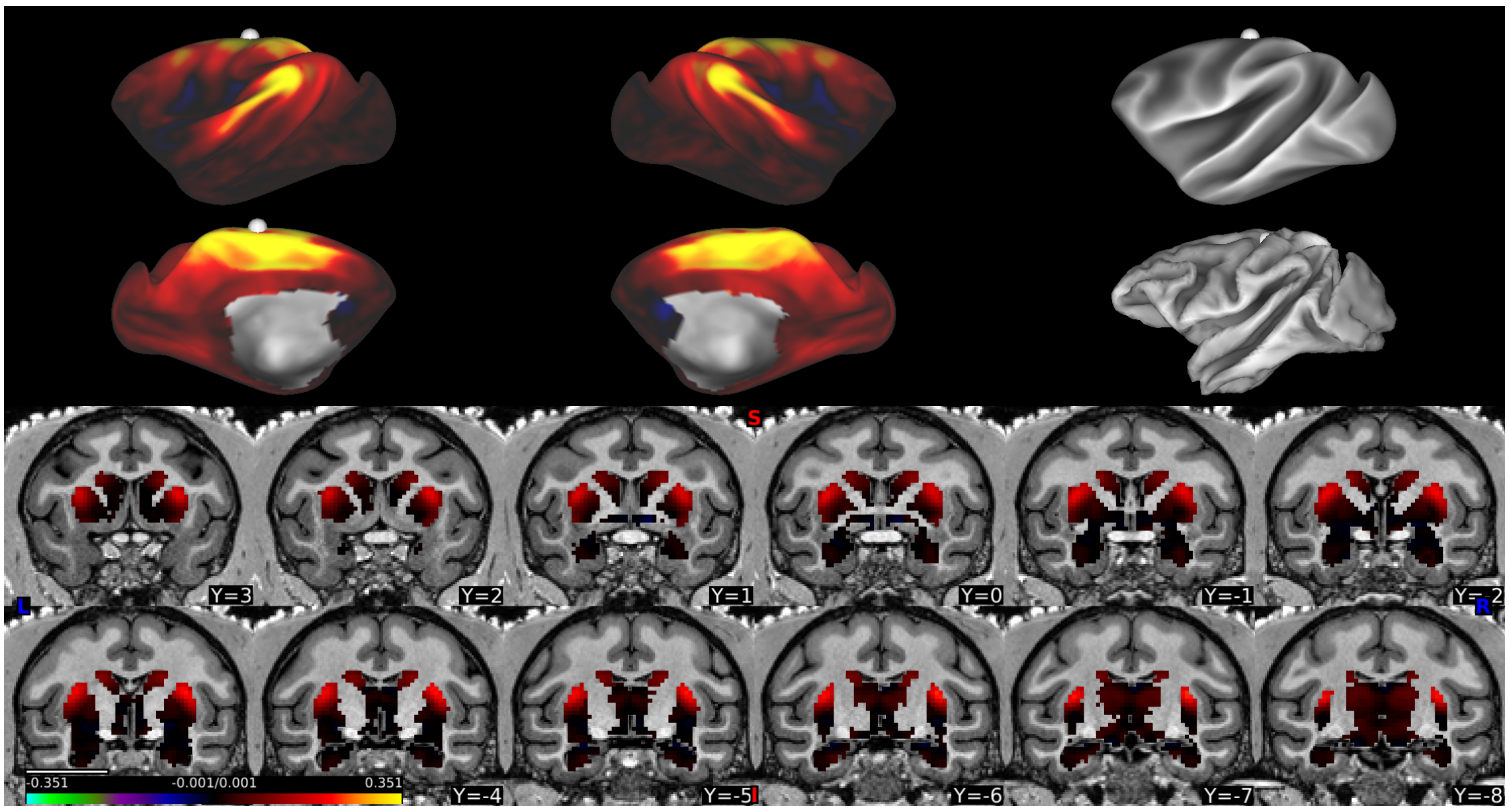

Movie S1. Seed-based functional connectivity maps in macaque monkeys

Group-average functional connectivity maps with 20 seeds in the left M1 are shown for the inflated left lateral (top row, left) and medial (second row, left) cortical surfaces, right lateral (top row, middle) and medial (second row, middle) cortical surfaces, and subcortical structures (third and bottom rows). The color map represents unthresholded connectivity (Fisher's z), scaled in the ± 98 percentile range for each seed condition. The seed location is indicated by the white spheres on the left inflated (top and second rows, left; top row, right) and middle thickness surfaces (second row, right). The cortical surface and T1w structural volume are from a representative subject (monkey FL).

Scale bar, 20 mm in bottom row.

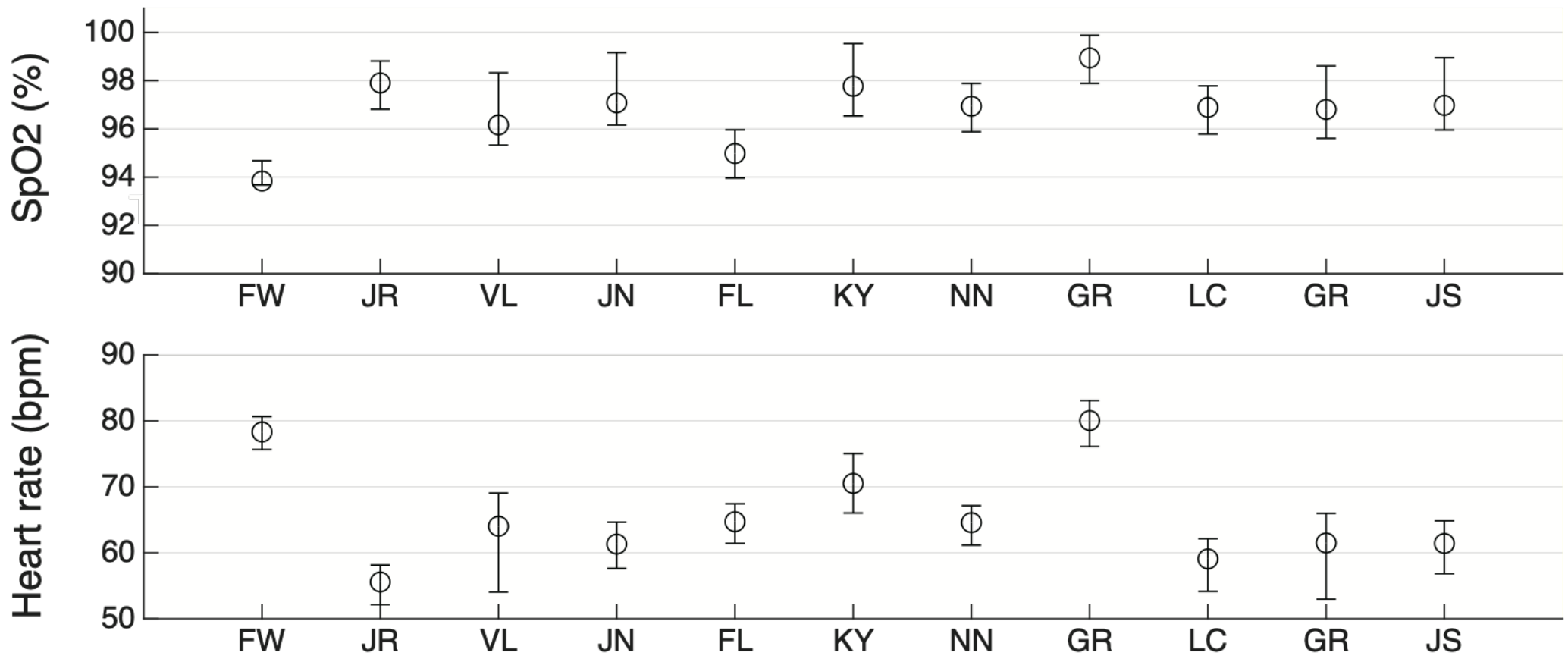

Figure S1. Physiological parameters during rs-fMRI acquisition.

Key physiological parameters recorded continuously during the rs-fMRI runs for individual macaques (top: peripheral oxygen saturation level [SpO_2]; bottom: heart rate). The circle and whiskers indicate the mean value and range (maximum and minimum values), respectively.

While there was inter-subject variability in the mean, the range was small in individual macaques, confirming that the anesthetic depth and physiological conditions were stable during data acquisition.

A

B

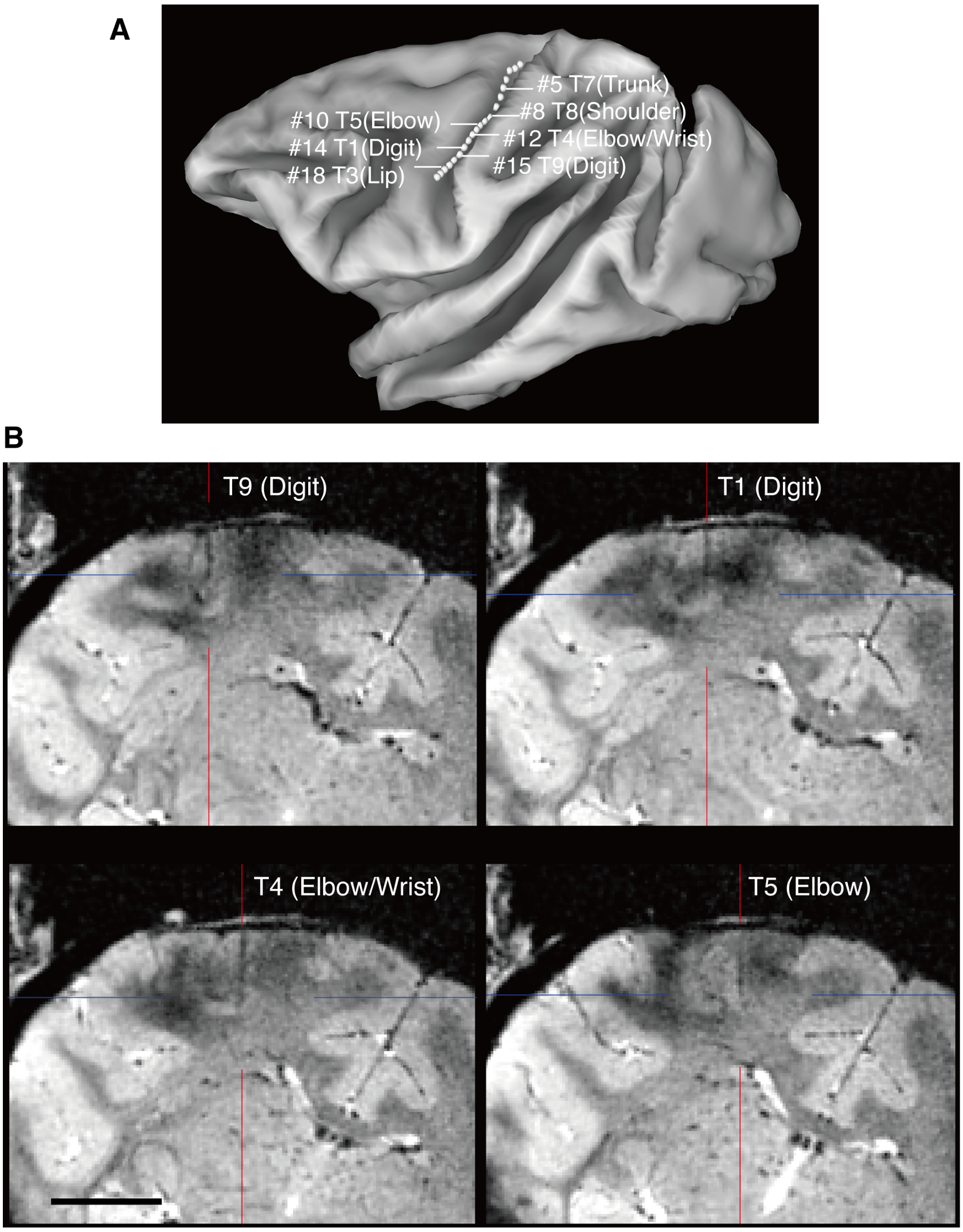

Figure S2. Twenty seed regions in the M1 and the corresponding somatotopic representations in a macaque monkey.

(A) Twenty seed regions located on the anterior bank of the left central sulcus, displayed on the left mid-thickness cortical surface (monkey FL). (B) A T2*w structural image of monkey FL acquired after a conventional somatotopic mapping using intracortical microstimulation. Tracks for injecting a viral vector—T9, T1, T4, and T5—are visible. These anatomical landmarks were used to guide the assignment of somatotopic representations to the seeds shown in (A). Scale bar, 10 mm in (B).

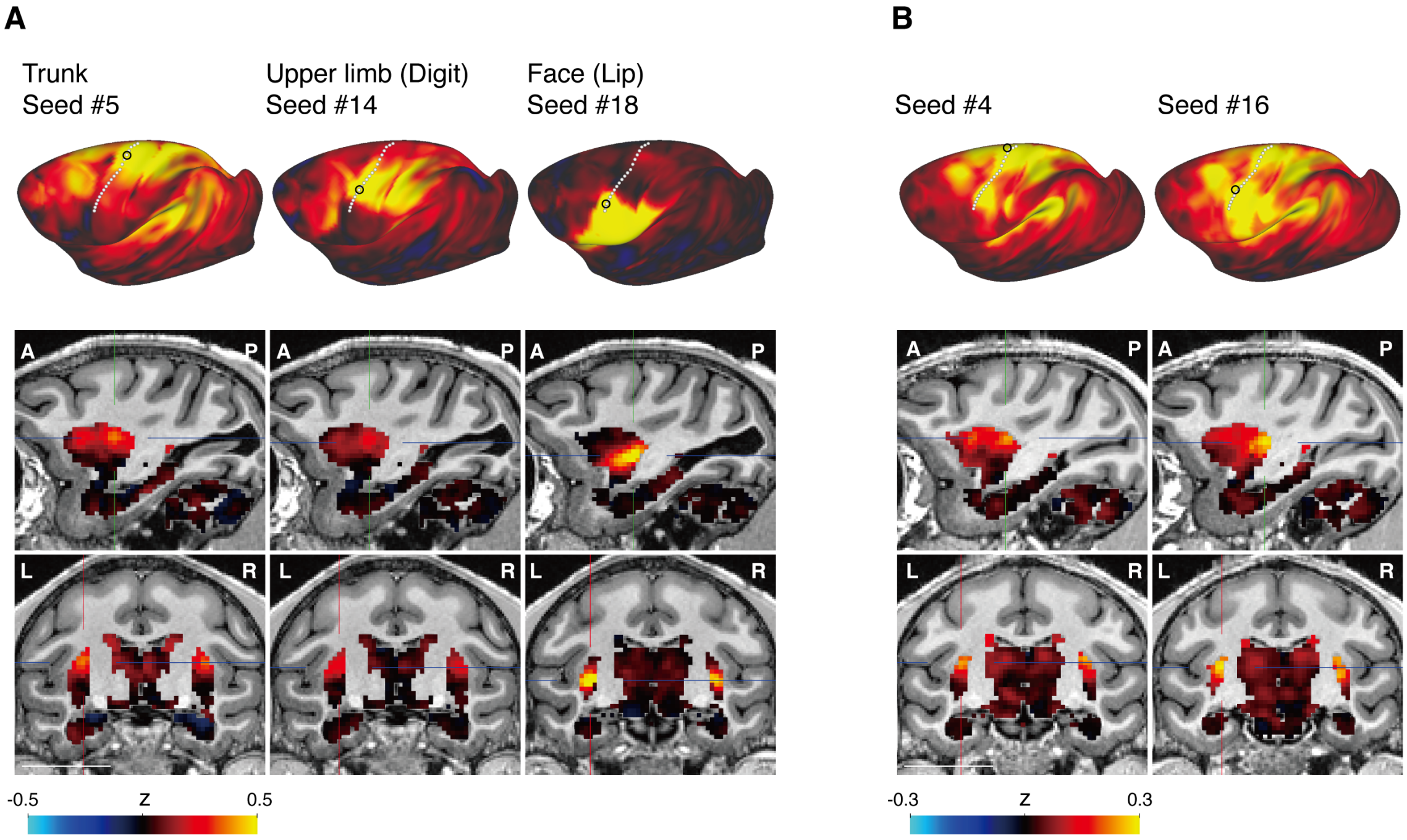

Figure S3. Seed-to-whole-brain functional connectivity maps in individual macaques

Whole-brain functional connectivity maps for two macaques (A: JR; B: SM). In (A), seeds were located at the M1 trunk (left), upper limb (middle) and face (right) regions, indicated with black circles on the cortical surfaces. The color map depicts unthresholded connectivity values (Fisher's z) in the left hemisphere (top) and subcortical structures (middle and bottom). The sagittal and coronal planes were centered on the peak of the connectivity in the left striatum. In (B), seeds were located at the putative inter-effector regions in the left M1. Cortical connectivity between the inter effector regions could be observed in this case. The cortical surface and T1w structural volume are from the respective subject. Scale bars, 20 mm in (A, B).

A: anterior, L: left, P: posterior, R: right.

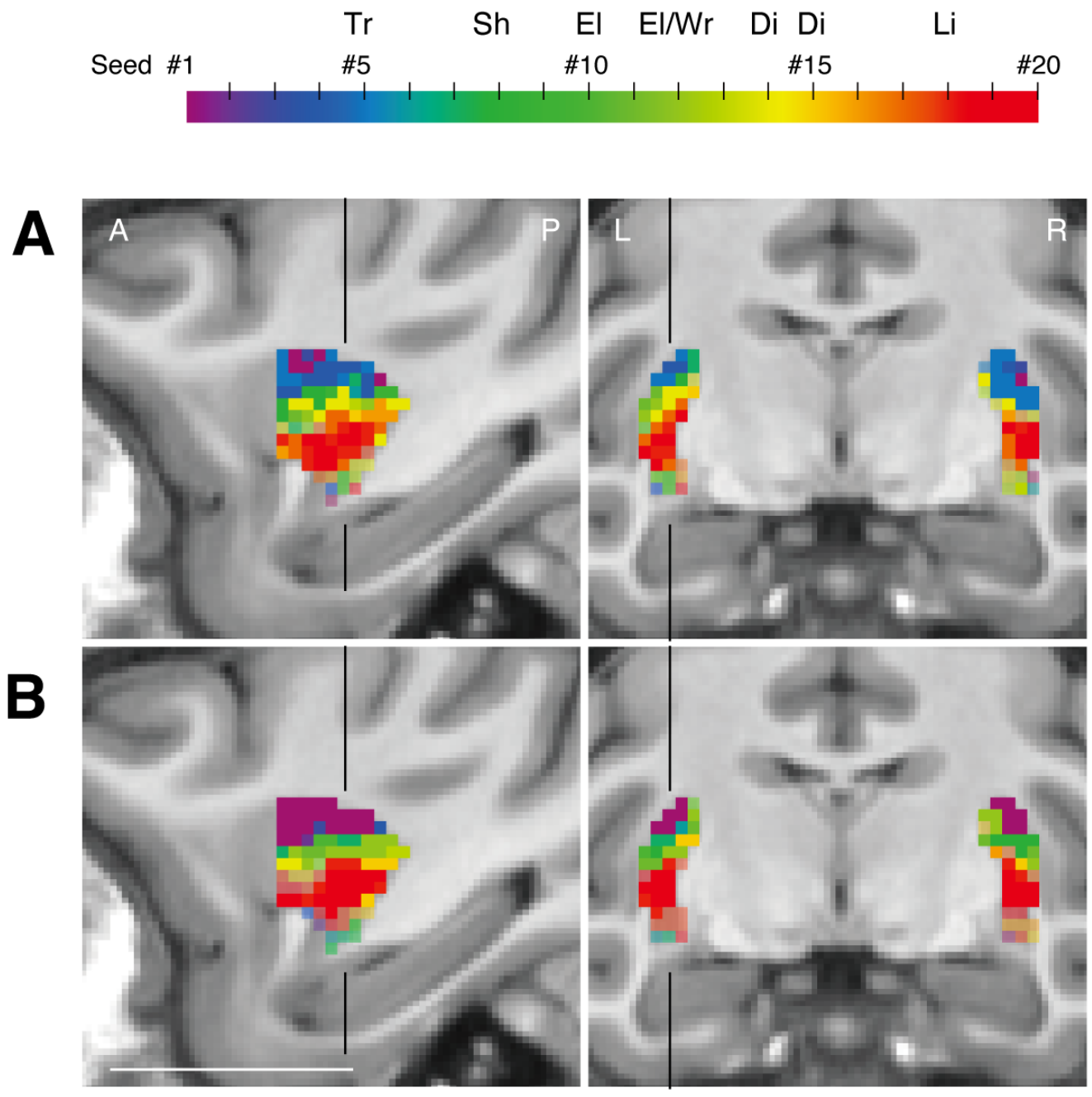

Figure S4. Volumetric somatotopic maps of the macaque posterior putamen derived from two subgroups

Map of the seed index with maximum normalized connectivity across 20 seeds derived from two subject subgroups, defined according to fMRI acquisition conditions: Group 1 subjects (A, N = 6) underwent 2 runs per subject, while Group 2 subjects (B, N = 5) underwent 4 runs. The violet-blue-green-yellow-red color map above (A) represents the seed index, ordered from lower limb to trunk, upper limb, and face. Voxel color was adjusted according to connectivity strength (100% saturation for the 100–75 percentile of Fisher's z, 75% for the 75–50 percentile, 50% for the 50–25 percentile, and 25% for the 25–0 percentile). Both maps show the same voxels as those shown in Fig. 2A. Structural images are group-average T1w volumes from the Yerkes19 template for macaques.

In both maps, a violet-blue-green-yellow-red gradient reflecting lower limb-trunk-upper limb-face somatotopy was observed in the dorsal to ventral direction. The gradients were significantly correlated ($r = 0.745$; $p < 10^{-6}$) between the two groups, despite the differences in acquisition conditions. Scale bar, 20 mm in (B).

Di: digit, El: elbow, Li: lip, Sh: shoulder, Tr: trunk, Wr: wrist.

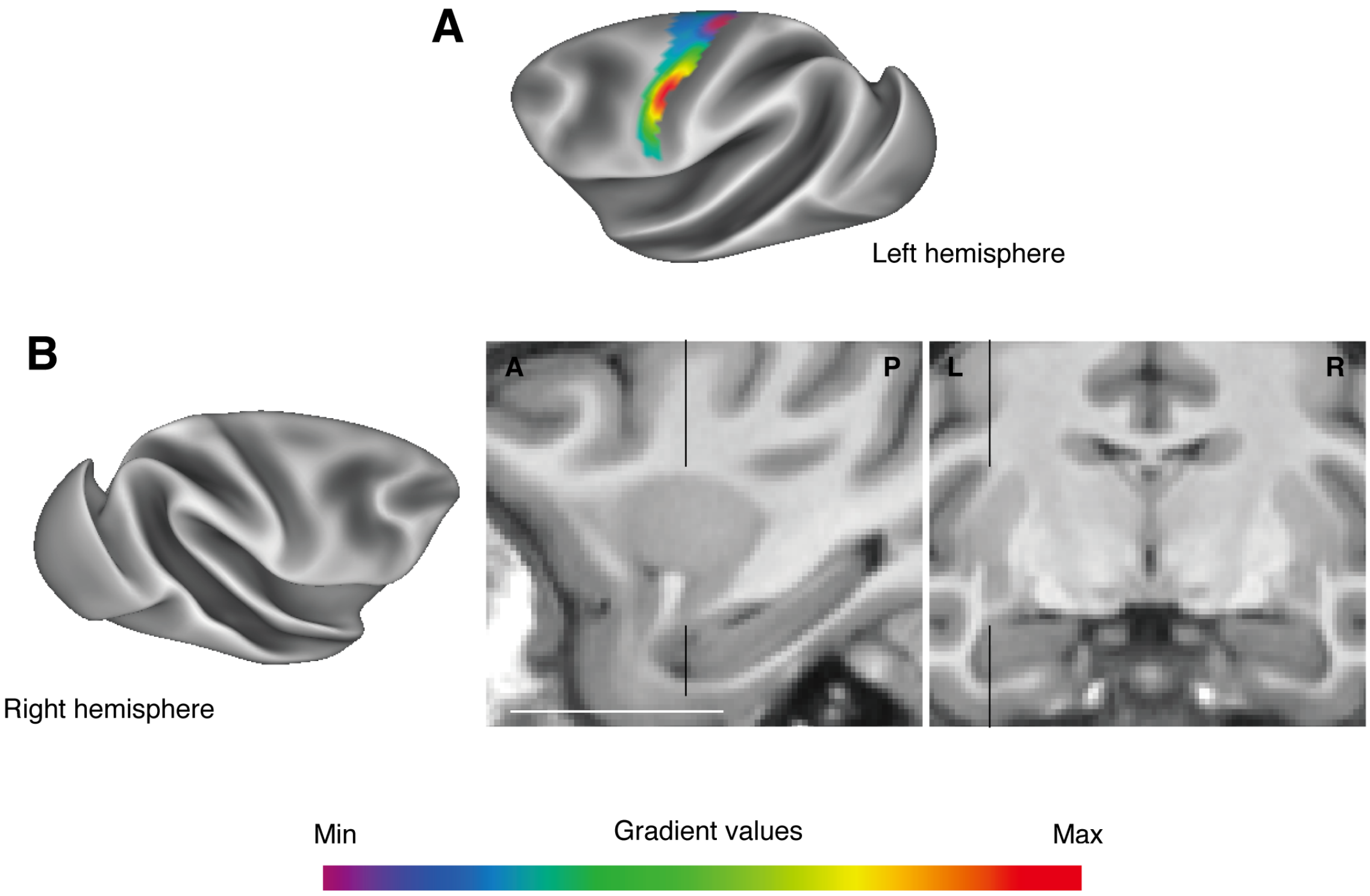

Figure S5. Connectopic mapping analysis with random time-series dataset

(A) First functional connectivity gradient component within the left M1 of macaque monkeys, estimated using connectopic mapping with random time series. The gradient was distinctly different from that estimated using the real dataset shown in Fig. 4A. (B) Projection of the first gradient component within the left M1 to the right cortical hemisphere (left) and to the subcortical structures (middle and right). The connectivity to the M1 vertices was all below the threshold (Fisher's z = 0.15), and there was no projection map in (B). The cortical surface is from monkey FL, and the T1w volume is from the Yerkes19 template. Scale bar, 20 mm in (B).
